# The most used questionnaires for evaluating satisfaction, usability, acceptance, and quality outcomes of mobile health

**DOI:** 10.1186/s12911-022-01764-2

**Published:** 2022-01-27

**Authors:** Sadrieh Hajesmaeel-Gohari, Firoozeh Khordastan, Farhad Fatehi, Hamidreza Samzadeh, Kambiz Bahaadinbeigy

**Affiliations:** 1 Medical Informatics Research Center, Institute for Futures Studies in Health, Kerman University of Medical Sciences, Kerman, Iran; 2 Department of Medical Informatics, Faculty of Medicine, Mashhad University of Medical Sciences, Mashhad, Iran; 3 Centre for Health Services Research, The University of Queensland, Brisbane, Australia; 4 School of Psychological Sciences, Monash University, Melbourne, Australia; 5 Department of Health Information Sciences, Faculty of Management and Medical Information Sciences, Kerman University of Medical Sciences, Kerman, Iran; 6 Gastroenterology and Hepatology Research Center, Institute of Basic and Clinical Physiology Sciences, Kerman University of Medical Sciences, Kerman, Iran

**Keywords:** Mobile health, Questionnaire, Evaluation

## Abstract

**Background:**

Various questionnaires are used for evaluating satisfaction, usability, acceptance, and quality outcomes of mobile health (mHealth) services. Using the best one to meet the needs of an mHealth study is a challenge for researchers. Therefore, this study aimed to review and determine the frequently used questionnaires for evaluating the mentioned outcomes of mHealth services.

**Methods:**

The PubMed database was searched for conducting this review in April 2021. Papers that used a referenced questionnaire to evaluate the satisfaction, usability, acceptance, or quality outcomes of mHealth were included. The first author’s name, year of publication, evaluation outcome, and evaluation questionnaire were extracted from relevant papers. Data were analyzed using descriptive statistics.

**Results:**

In total, 247 papers were included in the study. Questionnaires were used for usability (40%), quality (34.5%), acceptance (8.5%), and satisfaction (4%) outcomes, respectively. System usability scale (36.5%), mobile application rating scale (35.5%), post study system usability questionnaire (6%), user mobile application rating scale (5%), technology acceptance model (4.5%), computer system usability questionnaire (2.5%), net promoter score (2%), health information technology usability evaluation scale (2%), the usefulness, satisfaction, and ease of use (1.5%), client satisfaction questionnaire (1.5%), unified theory of acceptance and use of technology (1.5%), questionnaire for user interaction satisfaction (1%), user experience questionnaire (1%), and after-scenario questionnaire (1%) were the most used questionnaires, respectively.

**Conclusion:**

Despite the existence of special questionnaires for evaluating several outcomes of mHealth, general questionnaires with fewer items and higher reliability have been used more frequently. Researchers should pay more attention to questionnaires with a goal-based design.

**Supplementary Information:**

The online version contains supplementary material available at 10.1186/s12911-022-01764-2.

## Background

In recent years, mobile phones have found a special role in people's daily lives because of their portability and availability. Mobile phones are also used in the healthcare field for different purposes [1]. The use of mobile and wireless communication technologies to improve disease management, medication adherence, medical decision-making, medical education, and research is named mobile health (mHealth) [2, 3]. mHealth includes the use of simple capabilities of a mobile device such as voice call and short messaging service (SMS) as well as more complex applications designed for medical, fitness, and public health purposes [4].

mHealth could help patients to monitor and control their health when they do not have access to healthcare providers [1]. Along with the potential benefits of mHealth, some factors such as perceived ease of use, perceived usefulness, content quality and accuracy, and consumer attitude can influence the use of this technology [5]. Therefore, evaluating mHealth services in terms of different aspects such as usability, user satisfaction and acceptance, and quality is important.

Usability is defined as “the extent to which a product can be used by specified users to achieve specified goals with effectiveness, efficiency, and satisfaction in a specified context of use” by ISO 9241-11 [6]. A review study showed that about 88% of the studies that evaluated the usability of mobile applications used the above-mentioned definition [7]. There are two general methods for usability evaluation, including user evaluation and expert inspection [8]. User satisfaction is defined as “the net feeling of pleasure or displeasure that results from aggregating all the benefits that a person hopes to receive from interaction with the information system” [9]. The Cambridge Dictionary defines acceptance as a “general agreement that something is satisfactory or right” [10]. In the technology acceptance lifecycle, acceptance is measured in both the initial and sustained use stages of mHealth services [11]. As Stoyanov et al. indicated in their study, the quality of mHealth applications is evaluated in different categories, including engagement, functionality, aesthetics, information quality, and subjective quality [12].

There are various methods for evaluating mHealth services, such as questionnaires, interviews, and observation [11, 13, 14]. Researchers use a variety of general and specified questionnaires for evaluating different aspects of mHealth services. Studies usually use previously designed questionnaires [15, 16] and sometimes design a new one with compliance to their needs [12, 17]. The validity and reliability of the used questionnaires are important in any scientific project.

Due to the existence of a large number of questionnaires, selecting and using the appropriate one to meet the needs of an mHealth study is a challenge for researchers. To the best of our knowledge, no study has reviewed and listed the most appropriate questionnaires for evaluating different outcomes of mHealth services including satisfaction, usability, acceptance, and quality. Therefore, this study aimed to review and introduce the frequently used questionnaires for evaluating the mentioned outcomes. The results of this study will help other investigations to select the appropriate goal-based questionnaire.

## Methods

### Database and date

PubMed database was searched for conducting this review study. The search was performed on 18 April 2021 without date restriction.

### Search strategy

We used three categories of keywords for setting the search strategy (Table 1). The keywords in each category were combined and searched by OR Boolean operator. Then the results of these searches were combined by AND Boolean operator for retrieving relevant papers. The search was conducted in the Title/Abstract search field and was filtered with English language.

**Table 1:** Keywords used for the search strategy

Keywords		
mhealth	Evaluation	Questionnaire
Mobile health	Assessment	Scale
Mobile app		"Surveys and Questionnaires"[MeSH Terms]
Mobile application		
Self-management app		

### Inclusion criteria

The following studies were included in the study:

- Original observational and interventional research papers in which a referenced questionnaire or a questionnaire that has been used at least two times in the studies was used to evaluate the satisfaction, usability, acceptance, and quality outcomes of mhealth.
- App review studies that used the Mobile Application Rating Scale (MARS) for the evaluation of mhealth applications.

### Exclusion criteria

The following studies were excluded from the study:

- Review, protocol, conference, and report papers
- Papers without full text
- Papers that did not use mhealth services
- Papers that did not evaluate satisfaction, usability, acceptance, and quality outcomes
- Papers that did not use a referenced questionnaire
- Papers that did not include details about the questionnaires used

### Paper selection

In the first stage, all the retrieved papers were reviewed based on title and abstract by two authors (S.H, F.Kh). Next, the same individuals assessed the full text of the selected papers. In the cases of disagreements, the opinion of the other author (K.B) was asked. Finally, a list of included papers was provided.

### Data extraction

The first author’s name, year of publication, evaluation outcome, and evaluation questionnaire were extracted from the included papers.

### Data analysis

Data were analyzed using descriptive statistics including frequency and frequency percentage.

## Results

**Fig. 1:**
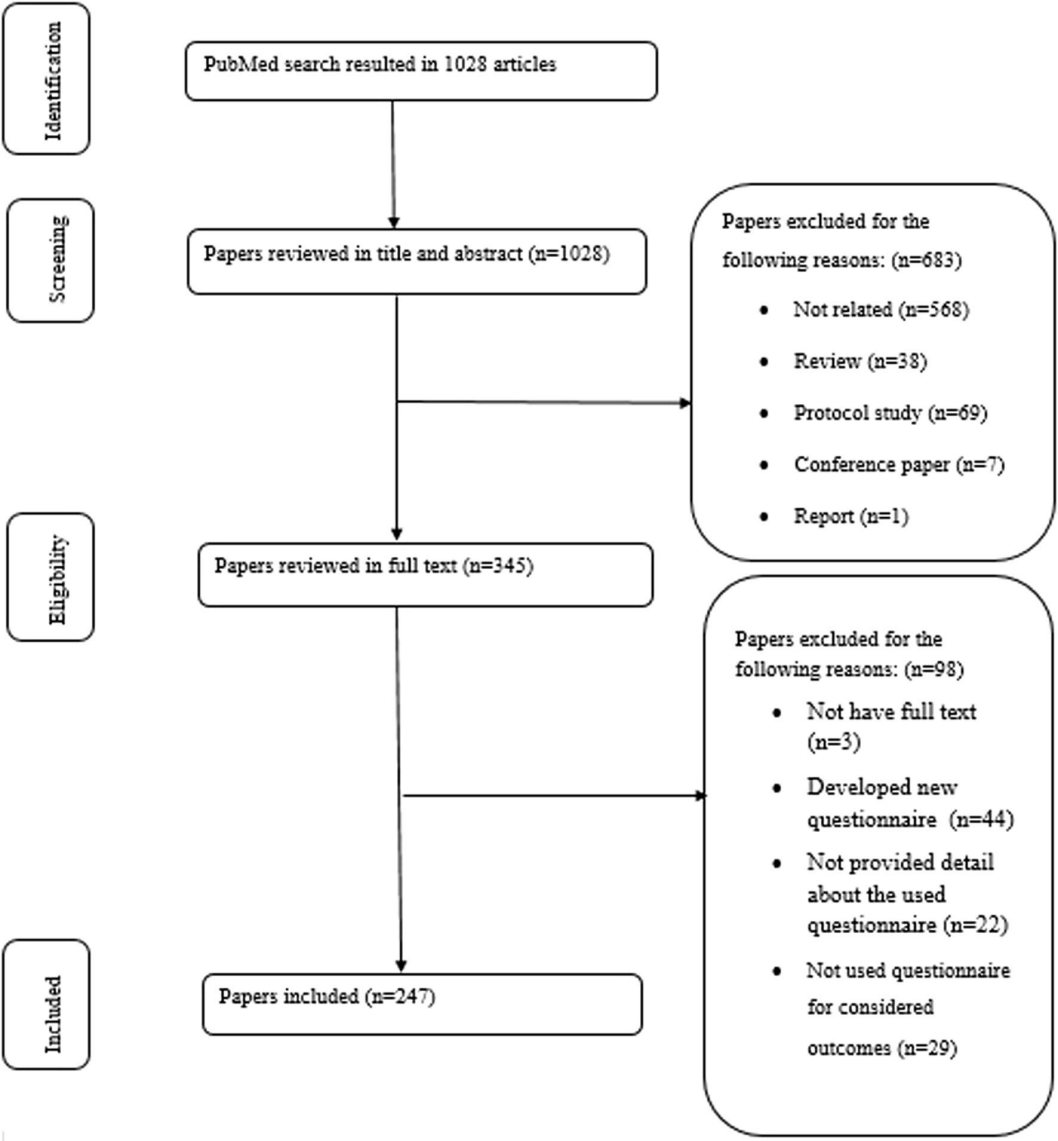
The process of finding and including the papers

Searching the PubMed database resulted in 1028 papers. The title and abstract of all these papers were screened. A total of 683 papers were excluded. After that, the full text of the 345 remaining papers was reviewed. Finally, 247 papers were included for extracting data (Fig. 1).

The extracted data from the included papers are presented in Additional file 1: Appendix 1. The main results were as follows:

### Year of publication

**Fig. 2:**
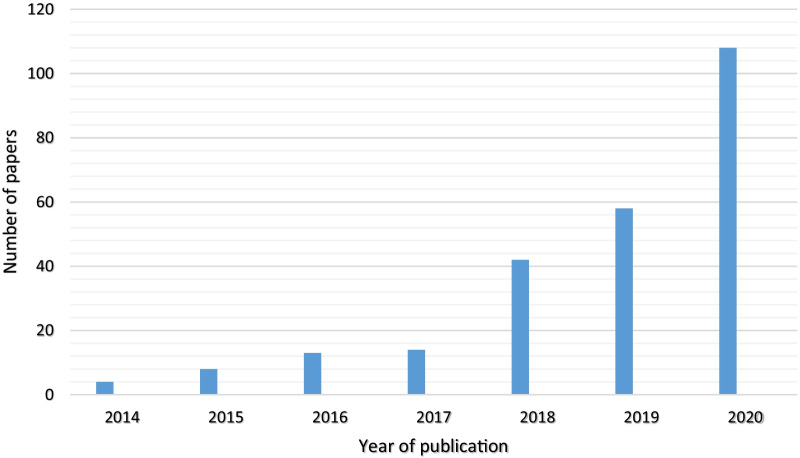
The number of papers based on the year of publication

The included papers have been published since 2014. The number of papers has increased since that time (Fig. 2). More than half of the papers (67%) were published in the last three years (2019, 2020, and 2021).

### Evaluation outcome

The evaluation outcomes in this study referred to usability, satisfaction, acceptance, and quality of mHealth services. Usability is the most evaluated outcome that was assessed by a questionnaire in the studies (n = 99, 40%). After that, quality (n = 85, 34.5%), acceptance (n = 21, 8.5%), and satisfaction (n = 10, 4%) were the most evaluated outcomes, respectively. The remaining papers evaluated more than one outcome, including usability and satisfaction (n = 10, 4%), usability and quality (n = 9, 3.5%), usability and acceptance (n = 9, 3.5%), satisfaction and quality (n = 3, 1%), and satisfaction and acceptance (n = 3, 1%).

### Evaluation questionnaire

The most used questionnaires (more than two times) for evaluating mHealth services are shown in Table 2. The other questionnaires have been used in 17 papers (7%). Forty-three (17.5%) papers used more than one questionnaire.

**Table 2:** The most frequently used questionnaires for evaluating mHealth services

Questionnaire	Frequency (%)	Description of the questionnaire
System usability scale (SUS)	90 (36.5)	Brooke et al. designed the SUS questionnaire. This questionnaire was introduced for evaluating the usability of electronic systems. The SUS questionnaire has ten items that are not set in any specific categories. These items were designed with a 5-point Likert scale. SUS has a high level of reliability with a coefficient alpha of 0.91 [18, 19]
Mobile application rating scale (MARS)	88 (35.5)	Stoyanov et al. presented MARS for evaluating the quality of mobile health applications. This tool contains 23 items in six categories, including engagement, functionality, aesthetics, information quality, and subjective quality. MARS also has one separate section that is named app-specific. The items of MARS have a 5-point scale [12]. The construct validity of the MARS was confirmed with the confirmatory factor analysis (the root mean square error of approximation = 0.074, Tucker–Lewis index = 0.922, confirmatory fit index = 0.940, standardized root mean square residual = 0.059). The reliability of this tool was also confirmed by Omega 0.79–0.93. The Concurrent validity of MARS showed that it correlates with ENLIGHT (*p* < 0.05) [20]
Post study system usability questionnaire (PSSUQ)	15 (6)	Lewis [21] developed the PSSUQ to evaluate user satisfaction with the system usability at the end of his study. The last version of this questionnaire has 16 questions in three sections, including system usefulness, information quality, and interface quality. These questions were designed with a 7-point Likert scale. The criterion validity of the PSSUQ showed a moderate correlation (r = 0.80) with other measures of user satisfaction. The reliability of PSSUQ is confirmed with a coefficient alpha of 0.96 [21]
user mobile application rating scale (uMARS)	12 (5)	Stoyanov et al. introduced the uMARS as an end-user version of the MARS in 2016. uMARS has 20 items in five sections, including engagement, functionality, aesthetics, information quality, and subjective quality. The perceived impact is an additional section in uMARS. The items of uMARS have a 5-point scale. The internal consistency of uMARS has been confirmed by Cronbach alpha = 0.90. The reliability of uMARS has been shown by the Intraclass Correlation Coefficient = 0.70 [22]
Technology acceptance model (TAM)	11 (4.5)	Davis [23] designed a questionnaire based on the TAM. TAM displays how users use and accept technology. The TAM questionnaire has 12 items that are arranged in two sections, including the perceived usefulness and perceived ease of use. This questionnaire showed high discriminant, convergent, and factorial validity. The reliability of the TAM has been confirmed with a Cronbach alpha of 0.98 for usefulness and 0.94 for ease of use sections [23]
Computer system usability questionnaire (CSUQ)	6 (2.5)	Lewis [24] designed the CSUQ to evaluate user satisfaction with the system usability. This questionnaire is similar to PSSUQ with different wording. CSUQ contains 19 items with a 7-point Likert scale. The reliability of CSUQ has been confirmed by Coefficient alpha more than 0.89 [24]
Net promoter score (NPS)	5 (2)	Reichheld presented the NPS to measure customer satisfaction. The only item of this tool is the following question: “How likely is it that you would recommend to a friend or colleague?” NPS has an 11-point scale [25]
Health information technology usability evaluation scale (Health-ITUES)	5 (2)	Yen et al. [26] developed Health-ITUES based on the Health IT Usability Evaluation Model (Health-ITUEM). This questionnaire has 20 items in four sections, including quality of work-life, perceived usefulness, perceived ease of use, and user control. The items of Health-ITUES have a 5-point Likert scale. The reliability and validity of this questionnaire confirmed with Cronbach’s alphas ranged from 0.81 to 0.95, and factor correlations ranged from 0.37 to 0.66 [26]
The usefulness, satisfaction, and ease of use (USE)	4 (1.5)	Lund et al. presented the USE questionnaire for assessing the usability of a system’s user interface. This questionnaire has 30 items in four sections, including usefulness, ease of use, ease of learning, and satisfaction. These items have a 7-point Likert scale [27]. The high correlations between the USE dimensions and the SUS (*r* between 0.60 and 0.82, *p* < 0.001) showed the validity of this questionnaire. The reliability of USE has been confirmed with Cronbach’s alpha = 0.98 [28]
Client satisfaction questionnaire (CSQ)	4 (1.5)	Larsen et al. [29] introduced the CSQ for evaluating user satisfaction with different services. This questionnaire has eight items with a 4-point scale. The reliability of CSQ has been confirmed with the Coefficient alpha = 0.93 [29]
Unified theory of acceptance and use of technology (UTAUT)	4 (1.5)	Venkatesh et al. [30] developed the UTAUT questionnaire based on the UTAUT model to assess user acceptance of technology. UTAUT consists of 16 items in four sections, including performance expectancy, effort expectancy, social influence, and facilitating conditions. The items are scaled with a 7-point Likert scale [30]
Questionnaire for user interaction satisfaction (QUIS)	3 (1)	Chin et al. [31] presented the QUIS to evaluate the usability of the system and interaction with the computer interface. QUIS has 27 items in five sections, including the overall satisfaction, screen, terminology and information, learning, and system capabilities. The items were designed with a 10-point scale. The factor analysis of this questionnaire showed satisfactory validity. The reliability of the QUIS was confirmed with Cronbach's alpha = 0.94 [31]
User experience questionnaire (UEQ)	3 (1)	Laugwitz et al. [32] introduced the UEQ for evaluating usability and user satisfaction. UEQ has 26 items in six sections, including attractiveness, perspicuity, efficiency, dependability, stimulation, and novelty. The items of this questionnaire are scaled using a 7-point scale. The factor analysis of this questionnaire showed satisfactory validity. The reliability of the UEQ was confirmed with Cronbach's alpha more than 0.71 [32]
After-scenario questionnaire (ASQ)	3 (1)	Lewis [33] designed the ASQ for evaluating user satisfaction in scenario-based usability testing. This questionnaire has three items with a 7-point graphical scale. The factor analysis of this questionnaire showed satisfactory validity. The Concurrent validity of the ASQ showed that it correlates with the scenario completion data (*p* < 0.01) The reliability of the ASQ was confirmed with the Coefficient alpha more than 0.90 [33]
mHealth app usability questionnaire (MAUQ)	2 (0.5)	Zhou et al. [17] developed the MAUQ for assessing the usability of interactive and standalone mHealth applications. The MAUQ for interactive applications has 21 items in three sections, including ease of use and satisfaction, system information arrangement, and usefulness. However, the MAUQ for standalone applications has 18 items in three sections including ease of use, interface and satisfaction, and usefulness. All items have a 7-point scale. The factor analysis of this questionnaire showed acceptable validity. The criterion and construct validity of MAUQ showed that it correlates with the PSSUQ (r = 0.8448) and the SUS (r = 0.6425). The reliability of the MAUQ was confirmed with Cronbach's alpha more than 0.80 [17]
Game experience questionnaire (GEQ)	2 (0.5)	Poels et al. [34] presented the GEQ for evaluating the satisfaction of digital game users. This questionnaire has 42 items with a 5-point scale. The factor analysis of this questionnaire showed acceptable validity. The reliability of the GEQ was confirmed with Cronbach's alpha more than 0.70 [34]
The perceived ease of use and usefulness questionnaire	2 (0.5)	Davis [23] designed the Perceived Ease of Use and Usefulness Questionnaire. This questionnaire has 12 items that are arranged in two sections, including perceived usefulness and perceived ease of use. All items are scaled with a 7-point scale. This questionnaire showed high discriminant, convergent, and factorial validity. The reliability of The Perceived Ease of Use and Usefulness Questionnaire has been confirmed with a Cronbach alpha more than 0.94 [23]

## Discussion

This study was performed to review the most frequently used questionnaires for evaluating satisfaction, usability, acceptance, and quality outcomes of mHealth services. Usability is the most evaluated outcome in the mHealth studies. SUS, PSSUQ, and CSUQ were the top three most used questionnaires for evaluating the usability of mHealth services, respectively. The two most used questionnaires for evaluating the quality of mHealth applications were MARS and uMARS. In addition, TAM and UTAUT were the most used questionnaires for measuring the user acceptance of mHealth services. The three most used questionnaires for evaluating user satisfaction were NPS, CSQ, and GEQ.

### Usability evaluation questionnaires

The present study showed that SUS questionnaire had been used much more than similar questionnaires such as PSSUQ and CSUQ in evaluating the usability of mHealth services. SUS is a general questionnaire that is used for evaluating the usability of electronic systems such as mobile devices. Compared with other questionnaires such as CSUQ, SUS is a quicker tool for judging the perceived usability of systems because it has fewer items with less scale pointing. This questionnaire also includes a question regarding the satisfaction of the user with the digital solution. The satisfaction evaluation questionnaires focus on tools that evaluate only this outcome, but it is also contained in the usability outcome [18, 19]. Because of these features and its reproducibility, reliability, and validity, researchers and evaluators of mHealth services have frequently used the SUS questionnaire. Another study that reviewed the most used questionnaires for evaluating telemedicine services also showed that SUS is the most used general questionnaire after the Telehealth Usability Questionnaire (TUQ), which is a specific questionnaire for evaluating the usability of telemedicine systems [35].

Although MAUQ was specifically designed for evaluating the usability of mHealth applications and considered both interactive and standalone mHealth applications [17], it was rarely used in the studies that were included in our review. This lack of use might be due to the fact that MAUQ was introduced 2 years ago, and researchers are less familiar with this questionnaire. It is recommended that researchers and evaluators of mHealth services use such questionnaires that were specifically designed for evaluating these services.

### Quality evaluation questionnaires

MARS and its user version (uMARS) were the most used questionnaires for assessing the quality of mHealth applications. To use MARS for evaluating mHealth applications, users should be professional in mHealth. Because of this limitation, uMARS was designed to be administered by end-users without special expertise. The importance of the quality and reliability of information and content provided in mHealth applications and the impact that this content has on people's health led to the design of MARS [12]. MARS prompted researchers to look at another consequence of mHealth, which significantly impacts the practical and safe use of mHealth applications. This issue has led to the use of these questionnaires in many studies.

### Acceptance evaluation questionnaires

This study revealed that TAM and UTAUT were the most used questionnaires for measuring mHealth acceptance. These questionnaires were derived from two models with the same name. Generally, TAM and UTAUT are the most used acceptance models in health informatics because of their simplicity [36]. Both models focus on the usefulness and easy use of technology. Since UTAUT derives from eight models such as TAM, it evaluates two additional factors, including social environment and organizational infrastructure, that may impact the adoption of the new technology [36]. However, since TAM and UTAUT have not been developed in healthcare settings, different emotional, organizational, and cultural factors that may influence technology acceptance in healthcare settings are not covered by these two questionnaires [23, 30]. Therefore, researchers in health informatics would better design the acceptance questionnaire based on the objective systems.

### Satisfaction evaluation questionnaires

The present research revealed that NPS is the most widely used tool for measuring the satisfaction of m-Health users. NPS is a very small tool for evaluating client satisfaction. This tool only has one question [25]. The fact that this scale has only one item has probably contributed to its wide use. It should be taken into account that a single question cannot identify the various factors that affect user satisfaction with a service. After NPS, CSQ and GEQ were the most used questionnaires in reviewed articles. CSQ has two characteristics that may affect its usage. The first one is that it considers the quality of different aspects, such as procedure, environment, staff, service, and outcome. The second characteristic is that with this comprehensiveness, this questionnaire has only eight items [29]. Studies that used mobile-based games to provide mHealth services used GEQ [37, 38] because it is a specific, comprehensive, and practical questionnaire that measures game user satisfaction [34]. Melin et al. presented a questionnaire for assessing the satisfaction of mHealth applications users [39]. However, none of the papers included in our study used this questionnaire because this is a new tool, and researchers are less familiar with it. It is recommended that researchers in mHealth, use this specific questionnaire in their future studies.

### Evaluation outcomes

Most of the included papers evaluated the usability of mHealth services using a questionnaire. Usability is a critical issue that affects willingness to use a system. Therefore, it is essential to evaluate this outcome in different phases of system development. The questionnaire is the most used method for evaluating the usability outcome of a mobile application because of its simpleness in terms of accomplishment and data analysis [17]. A review study also showed that the usability of mHealth applications is mostly assessed using a questionnaire [40]. Another study revealed that questionnaires were mostly used for evaluating the user satisfaction outcome of telemedicine [35]. The differences between the results of our research and those of this study may be due to the fact that mHealth services are mostly presented with an application; therefore, evaluating the user interface of the application is very important and should be considered for effective use [40].

### Limitations

To the best of our knowledge, this is the first study that reviewed the most used questionnaires for evaluating the satisfaction, usability, acceptance, and quality outcomes of mHealth services. Nevertheless, this study has some limitations. We only searched the PubMed database to retrieve relevant papers. Also, we restricted our search to the Title/Abstract field. Moreover, we excluded review papers and only included the app review studies that use MARS. These limitations may have led to the missing of some papers from our study.

## Conclusion

This study showed that usability and quality were the most frequently considered outcomes in the mHealth field. Since user acceptance and satisfaction with mHealth services lead to more engagement in using these applications, they should be more considered. Although there is a questionnaire that is specifically designed for measuring several mHealth outcomes, general questionnaires such as SUS, PSSUQ, TAM, CSUQ, Health-ITUES, the USE, CSQ, UTAUT, QUIS, UEQ, and ASQ are mostly used for evaluating mHealth services. Moreover, the results showed that researchers prefer to use questionnaires with high reliability and fewer items. Therefore, when selecting the best-fitted questionnaires for evaluating different outcomes of mHealth services, it is better to pay more attention to the reliability and the number of questions and items.

## Supplementary Information

**Additional file 1**. The extracted data from the included papers.

## Data Availability

Not applicable.

## Abbreviations

mhealth: Mobile health
ISO: International Organization for Standardization
MARS: Mobile application rating scale
SUS: System usability scale
PSSUQ: Post study system usability questionnaire
uMARS: User mobile application rating scale
TAM: Technology acceptance model
CSUQ: Computer system usability questionnaire
NPS: Net promoter score
Health-ITUES: Health information technology usability evaluation scale
USE: Usefulness, satisfaction, and ease of use
CSQ: Client satisfaction questionnaire
UTAUT: Unified theory of acceptance and use of technology
QUIS: Questionnaire for user interaction satisfaction
UEQ: User experience questionnaire
ASQ: After-scenario questionnaire
MAUQ: MHealth app usability questionnaire
GEQ: Game experience questionnaire
TUQ: Telehealth usability questionnaire
SUTAQ: Service user technology acceptability questionnaire
