# Supplementary material for: The most used questionnaires for evaluating satisfaction, usability, acceptance, and quality outcomes of mobile health
Source: BMC Med Inform Decis Mak. 2022 Jan 27;22:22. doi: 10.1186/s12911-022-01764-2 (PMC8793175; doi:10.1186/s12911-022-01764-2)
Supplement: Supplementary file 1 — Additional file 1. The extracted data from the included papers. [file 12911_2022_1764_MOESM1_ESM.docx]

| **Reference** | **Publication year** | **Evaluation outcome** | **Evaluation questionnaire** |
| --- | --- | --- | --- |
| Alanzi, et al (1) | 2016 | Usability | Questionnaire for User Interaction Satisfaction (QUIS)(2) |
| Alhuwail, et al(3) | 2019 | Quality | Mobile Application Rating Scale (MARS)(4) |
| Alnasser, et al(5) | 2018 | Usability | System Usability Scale (SUS)(6) |
| Alshathri, et al(7) | 2020 | Quality | MARS(4) |
| Amor-García, et al(8) | 2020 | Quality | MARS(4) |
| Appleton, et al(9) | 2019 | Usability | SUS(6) |
| Argent, et al(10) | 2019 | Usability, Quality | SUS(6), The user version of the MARS (uMARS)(11) |
| Athilingam, et al(12) | 2018 | Usability | MARS(4) |
| Bahadori, et al(13) | 2020 | Quality | MARS(4) |
| Bailon, et al(14) | 2019 | Usability | SUS(6) |
| Bakogiannis, et al(15) | 2021 | Usability, Quality | The Post-Study System Usability Questionnaire (PSSUQ)(16), MARS(4) |
| Balsa, et al(17) | 2020 | Usability | Portuguese version of SUS(18) |
| Baltaxe, et al(19) | 2020 | Usability, Satisfaction | SUS(6), Net Promoter Score (NPS)(20), Nijmegen Continuity Questionnaire(21), Person-Centred Coordinated Care Experience Questionnaire(22) |
| Bardus, et al(23) | 2020 | Quality | Arabic Version of the MARS(23) |
| Barteit, et al(24) | 2019 | Acceptance | TAM2(25), DeLone and McLean’s IS success mode(26) |
| Beatty, et al(27) | 2018 | Usability | SUS(6) |
| Belarmino, et al(28) | 2018 | Satisfaction | Questionnaire has been used in previous study(29) |
| Bente, et al(30) | 2021 | Usability | Dutch User Experience Questionnaire (UEQ-Dutch)(31) |
| Bentley, et al(32) | 2020 | Usability | SUS(6) |
| Biello, et al(33) | 2021 | Acceptance | SUS(6) |
| Boyle, et al(34) | 2018 | Quality | MARS(4) |
| Brinkel, et al(35) | 2017 | Usability | SUS(6) |
| Broekhuis, et al(36) | 2019 | Satisfaction, Usability | After-Scenario Questionnaire (ASQ)(37), SUS(6) |
| Brown, et al(38) | 2018 | Quality | MARS(4) |
| Cervera, et al(39) | 2018 | Usability | PSSUQ(16) |
| Chang, et al(40) | 2019 | Usability | SUS(6) |
| Chapman, et al(41) | 2018 | Quality | MARS(4) |
| Chávez, et al(42) | 2019 | Usability | TAM(25) |
| Chen, et al(43) | 2021 | Quality | MARS(4) |
| Chen, et al(44) | 2017 | Acceptance | TAM(25) |
| Cheng, et al(45) | 2020 | Quality | MARS(4) |
| Cho, et al(46) | 2018 | Usability | PSSUQ(16) |
| Choi, et al(47) | 2020 | Usability | SUS(6) |
| Choi, et al(48) | 2021 | Quality | MARS(4) |
| Choi, et al(49) | 2018 | Quality | MARS(4) |
| Chung, et al(50) | 2021 | Usability | The Usefulness, Satisfaction, and Ease of Use (USE) Questionnaire(51) |
| Clebone, et al(52) | 2019 | Usability | SUS(6) |
| Corazza, et al(53) | 2020 | Usability | UEQ(54) |
| Cordova, et al(55) | 2018 | Acceptance | Modified Session Evaluation Form (SEF)(56), Client Satisfaction Questionnaire-8 (CSQ-8)(57) |
| Costa Stutzel, et al(58) | 2019 | Satisfaction, Usability | Marked Semantic Differential Scale(59), SUS(6) |
| Davalbhakta, et al(60) | 2020 | Quality | MARS(4) |
| Davis, et al(61) | 2019 | Quality | uMARS(11) |
| Day, et al(62) | 2019 | Usability | NPS(20) |
| de Batlle, et al(63) | 2020 | Satisfaction | NPS(20), SUS(6) |
| De Cock, et al(64) | 2018 | Satisfaction | Game Experience Questionnaire (GEQ)(65) |
| de Dicastillo, et al(66) | 2019 | Usability | SUS(6) |
| Deady, et al(67) | 2020 | Quality, Usability | uMARS(11), USE (51) |
| Deady, et al(68) | 2018 | Acceptance, Usability | Questionnaire used in previous study(69) |
| Devan, et al(70) | 2019 | Quality | MARS(4) |
| Díaz, et al(71) | 2021 | Usability | SUS(6) |
| Dittrich, et al(72) | 2020 | Quality | German MARS(73) |
| Dunsmuir, et al(74) | 2019 | Usability | Computer System Usability Questionnaire (CSUQ)(75) |
| Eapen, et al(76) | 2015 | Usability | SUS(6) |
| Ehrler, et al(77) | 2018 | Acceptance | Modified the Unified Theory  of Acceptance and Use of Technology (UTAUT)(78) |
| English, et al(79) | 2016 | Satisfaction | Modified CSUQ(75) |
| Escriche-Escuder, et al(80) | 2020 | Quality | MARS(4) |
| Farzandipour, et al(81) | 2019 | Usability | QUIS(2) |
| Fazzino, et al(82) | 2018 | Usability | CSUQ(75) |
| Ferguson, et al(83) | 2020 | Usability, Quality | SUS(6), uMARS(11) |
| Fijačko, et al(84) | 2020 | Quality | uMARS(11) |
| Floryan, et al(85) | 2020 | Quality | MARS(4) |
| Fraynt, et al(86) | 2018 | Quality | MARS(4) |
| Fu, et al(87) | 2016 | Usability | The Perceived Ease of Use and Usefulness Questionnaire(25), PSSUQ(16) |
| Fuller-Tyszkiewicz, et al(88) | 2018 | Usability | SUS(6) |
| Furlong, et al(89) | 2018 | Quality | MARS(4) |
| Galve Villa, et al(90) | 2020 | Usability, Acceptance | SUS(6), modified TAM(25) |
| Gamwell, et al(91) | 2021 | Quality | MARS(4) |
| Gandhi, et al(92) | 2018 | Usability | SUS(6) |
| Gannon, et al(93) | 2020 | Usability | Health Information Technology Usability Evaluation Scale (Health-ITUES)(94), PSSUQ(16) |
| Garzo,et al(95) | 2018 | Usability | SUS(6) |
| Georgsson, et al(96) | 2016 | Usability | Questionnaire has been used in previous study(97) |
| Georgsson, et al(98) | 2016 | Satisfaction | SUS(6) |
| Ghanbari, et al(99) | 2017 | Usability | Questionnaire has been used in previous study(100) |
| Gill, et al(101) | 2019 | Usability | PSSUQ(16) |
| Gong, et al(102) | 2020 | Quality | MARS(4) |
| Grainger, et al(103) | 2017 | Quality | MARS(4) |
| Grainger, et al(104) | 2020 | Usability | SUS(6) |
| Grasaas, et al(105) | 2019 | Usability | SUS(6) |
| Grindrod, et al(106) | 2014 | Usability | SUS(6) |
| Ha, et al(107) | 2016 | Usability | CSUQ(75) |
| Hafiz, et al(108) | 2020 | Usability | MARS(4) |
| Hemmings, et al(109) | 2021 | Usability | SUS(6) |
| Herbuela, et al(110) | 2020 | Quality | MARS(4) |
| Hill, et al(111) | 2021 | Usability | Simplified SUS for Cognitively Impaired and Older Adults(112) |
| Ho, et al(113) | 2020 | Quality | MARS(4) |
| Hoffmann, et al(114) | 2020 | Quality | MARS(4) |
| Holden, et al(115) | 2020 | Usability | SUS(6) |
| Hsieh, et al(116) | 2018 | Usability | SUS(6) |
| Hyatt, et al(117) | 2020 | Quality | MARS(4) |
| Islam, et al(118) | 2020 | Usability | SUS(6) |
| Ithnin, et al(119) | 2017 | Usability | SUS(6) |
| Jacobson, et al(120) | 2017 | Usability, Satisfaction | SUS(6), Questionnaire has been used in previous study(121) |
| Jain, et al(122) | 2019 | Quality, Satisfaction | MARS(4), Questionnaire has been used in previous study(123) |
| Jeon, et al(124) | 2015 | Usability | PSSUQ(16) |
| Ji(125) | 2015 | Usability | Modified TAM(25) |
| Ji, et al(126) | 2020 | Usability | SUS(6) |
| Jones, et al(127) | 2020 | Quality | MARS(4) |
| Jonker, et al(128) | 2021 | Acceptance, Usability | NPS(20), SUS(6) |
| Jovičić, et al(129) | 2021 | Quality, Usability | MARS(4), SUS(6) |
| Jovičić, et al(130) | 2018 | Quality | MARS(4) |
| Juengst, et al(131) | 2015 | Usability, Satisfaction | Telehealth Usability Questionnaire (TUQ)(132) |
| Jung, et al(133) | 2021 | Usability | ASQ(37) |
| Jupp, et al(134) | 2018 | Quality | MARS(4) |
| Kalhori, et al(135) | 2020 | Quality | MARS(4) |
| Kalz, et al(136) | 2014 | Usability | SUS(6) |
| Khowaja, et al(137) | 2020 | Usability | SUS(6) |
| Kim, et al(138) | 2015 | Usability | SUS(6) |
| Kim, et al(139) | 2020 | Quality | Smartphone App Evaluation Tool(140) |
| Kizakevich, et al(141) | 2018 | Usability | SUS(6) |
| Kizakevich(142) | 2014 | Usability | SUS(6) |
| Klingberg, et al(143) | 2018 | Usability | Health-ITUES(94) |
| Klingemann, et al(144) | 2020 | Usability, Quality | SUS(6), MARS(4) |
| Kooij, et al(145) | 2021 | Acceptance | UTAUT(78) |
| Kristjansdottir, et al(146) | 2020 | Usability | SUS(6) |
| Kuhns, et al(147) | 2021 | Usability, Satisfaction | Health-ITUES(94), PSSUQ(16), CSQ(57) |
| Kwan, et al(148) | 2019 | Quality | MARS(4) |
| Lambert, et al(149) | 2017 | Quality | MARS(4), The Silberg scale(150) |
| Langlet, et al(151) | 2020 | Usability | SUS(6) |
| Langlet, et al(152) | 2021 | Usability | SUS(6) |
| Lawitschka, et al(153) | 2020 | Satisfaction | GEQ(65) |
| LeBeau, et al(154) | 2019 | Quality | uMARS(11) |
| Li, et al(155) | 2021 | Quality | MARS(4) |
| Li, et al(156) | 2019 | Quality | uMARS(11) |
| Liao, et al(157) | 2020 | Acceptance | Questionnaire has been used in previous study(158) |
| Lim, et al(159) | 2015 | Usability | CSUQ(75) |
| Lim, et al (160) | 2020 | Quality | MARS(4) |
| Liu, et al (161) | 2020 | Acceptance | SUS(6),CSQ-8(57) |
| Liu, et al (162) | 2018 | Usability | PSSUQ(16), Nielsen’s Measurement Scales for Perceived Usefulness and Ease of Use, SUS(6), TAM(25) |
| Liu, et al(163) | 2021 | Quality | MARS(4) |
| Liu, et al(164) | 2020 | Usability | SUS(6) |
| Liu, et al(165) | 2020 | Usability | SUS(6) |
| Logsdon, et al(166) | 2019 | Quality | MARS(4) |
| Loh, et al(167) | 2018 | Usability, Satisfaction | SUS(6), Modified patient satisfaction survey that used in past study(168) |
| Lozano, et al(169) | 2019 | Quality, Satisfaction | MARS(4), NPS(20) |
| Lozano, et al(170) | 2020 | Satisfaction | Questionnaire has been used in previous study(171) |
| Lu, et al(172) | 2021 | Quality | MARS(4) |
| Luna, et al(173) | 2019 | Satisfaction, Usability | Satisfaction scale questionnaire has been used in previous study(174), Modified Korhonen’s heuristics(175) |
| Luo, et al(176) | 2020 | Usability, Acceptance | Modified PSSUQ(16), and the Acceptability e-Scale(177) |
| Luštrek, et al(178) | 2021 | Acceptance | UTAUT(78) |
| Lyzwinski, et al(179) | 2019 | Quality | MARS(4) |
| Macedo, et al(180) | 2020 | Satisfaction, Acceptance | SUS(6), TAM(25) |
| Maguire, et al(181) | 2020 | Acceptance | TAM for ehealth(25, 182) |
| Mandracchia, et al(183) | 2020 | Quality | MARS(4) |
| Mani, et al(184) | 2015 | Quality | MARS(4) |
| Marques, et al(185) | 2020 | Usability | Smartphone Usability Questionnaire(186) |
| Martin, et al(187) | 2019 | Quality | MARS(4) |
| Martin, et al(188) | 2019 | Quality | MARS(4) |
| Masterson, et al(189) | 2016 | Quality | MARS(4) |
| Mattson(190) | 2016 | Usability | IsoMetrics questionnaire(191) |
| Mauch, et al(192) | 2018 | Quality | MARS(4) |
| Mayoral, et al(193) | 2021 | Usability | SUS(6) |
| Meedya, et al(194) | 2020 | Usability, Acceptance | Questionnaire has been used in previous study(195) |
| Mehdi, et al(196) | 2020 | Quality | MARS(4) |
| Messner, et al(197) | 2020 | Quality | MARS(4) |
| Metelmann, et al(198) | 2018 | Usability | SUS(6) |
| Moeini, et al(199) | 2021 | Usability | CSUQ(75) |
| Mohamad Marzuki, et al(200) | 2019 | Usability | SUS(6) |
| Mohamad Marzuki, et al(201) | 2018 | Usability | SUS(6) |
| Mohseni Moallem Kolaei, et al(202) | 2021 | Usability | QUIS(2) |
| Monteiro-Guerra, et al(203) | 2020 | Usability, Quality | SUS(6), Satisfaction rating questionnaire (204), MARS(4) |
| Moradian, et al (205) | 2018 | Acceptance | Modified Telehealth Acceptance Measure(206) |
| Moral-Munoz, et al(207) | 2018 | Quality | MARS(4) |
| Morita, et al(208) | 2019 | Usability | SUS(6) |
| Moura, et al(209) | 2021 | Usability | 247 web usability guidelines(210) |
| Muhindo, et al(211) | 2021 | Acceptance | SUS(6), The Center for Health Care Evaluation Provider Satisfaction Questionnaire (CHCE-PSQ)(212) |
| Musgrave, et al(213) | 2020 | Quality | MARS(4) |
| Müssener, et al(214) | 2020 | Usability | SUS(6) |
| Newton, et al(215) | 2020 | Satisfaction, Usability | CSQ(57), SUS(6) |
| Nguyen, et al(216) | 2020 | Usability | SUS(6) |
| Nguyen, et al(217) | 2020 | Quality | MARS(4) |
| Olaoye, et al(218) | 2020 | Acceptance | Questionnaire has been used in previous study(219) |
| Ong, et al(220) | 2019 | Usability | The extended Short Feedback Questionnaire (eSFQ)(221), Perceived playability of the mobile  Apps from previous study(222), Playability and Usability  Questionnaire (PUQ)(223), uMARS(11) |
| O'Reilly, et al(224) | 2018 | Usability, Quality | SUS(6), uMARS(11) |
| Oyetunde, et al(225) | 2019 | Acceptance | TAM(25) |
| Pande, et al(226) | 2017 | Usability, Acceptance | SUS(6), TAM(25) |
| Peek, et al(227) | 2021 | Acceptance | uMARS(11) |
| Petersen, et al(228) | 2020 | Usability, Acceptance | SUS(6), USE(51) |
| Phillips, et al(229) | 2021 | Quality | MARS(4) |
| Portenhauser, et al(230) | 2021 | Quality | MARS(4) |
| Pulantara, et al(231) | 2018 | Usability | SUS(6), Modified Telerehabilitation Usability Questionnaire (TUQ)(232) |
| Quan, et al(233) | 2020 | Acceptance | Technology Readiness Index (TRI)(234) |
| Quinn, et al(235) | 2019 | Quality, Usability | The Modified MARS(4), SUS(6) |
| Rajkumar, et al(236) | 2020 | Usability | SUS(6) |
| Ramsey, et al(237) | 2019 | Quality | MARS(4) |
| Regmi, et al(238) | 2017 | Quality | MARS(4) |
| Reyes, et al(239) | 2016 | Quality | MARS(4) |
| Richardson, et al(240) | 2019 | Quality | MARS(4) |
| Roberts, et al(241) | 2021 | Quality | Adapted MARS (A-MARS)(4) |
| Rodante, et al(242) | 2020 | Acceptance | The User Experience Questionnaire short version (UEQ-s)(243) |
| Rodrigues, et al(244) | 2020 | Quality | MARS(4) |
| Romero, et al(245) | 2019 | Quality | MARS(4) |
| Rudolf, et al(246) | 2019 | Satisfaction, Quality | SUS(6), The AttrakDiff questionnaire(247) |
| Salazar, et al(248) | 2018 | Quality | MARS(4) |
| Salehinejad, et al(249) | 2020 | Quality | MARS(4) |
| Sandhu, et al(250) | 2019 | Usability | SUS(6), General posttest  questionnaire(251) |
| Santo, et al(252) | 2016 | Quality | MARS(4) |
| Satre, et al(253) | 2017 | Usability | SUS(6) |
| Scherr, et al(254) | 2021 | Usability | SUS(6) |
| Schmidt, et al(255) | 2020 | Usability | SUS(6) |
| Schnall, et al(256) | 2018 | Usability | Health-ITUES(94), PSSUQ(16) |
| Schoeppe, et al(257) | 2017 | Quality | MARS(4) |
| Sedlmayr, et al(258) | 2018 | Usability | SUS(6) |
| Segura-Sampedro, et al(259) | 2017 | Satisfaction | Telemedicine Satisfaction Questionnaire (TSQ)(260) |
| Sengupta, et al(261) | 2020 | Usability | SUS(6) |
| Sereda, et al(262) | 2019 | Quality | MARS(4) |
| Setiawan, et al(263) | 2019 | Usability | PSSUQ(16) |
| Sevilla-Gonzalez, et al(264) | 2020 | Usability | SUS(6) |
| Shalan, et al(265) | 2018 | Usability | modified SUS(266) |
| Shang, et al(267) | 2019 | Quality | MARS(4) |
| Sharif, et al(268) | 2019 | Quality | MARS(4) |
| Siddique, et al(269) | 2019 | Quality | MARS(4) |
| Sood, et al(270) | 2020 | Usability | mHealth App Usability Questionnaire (MAUQ)(271) |
| Soomro, et al(272) | 2019 | Quality | Modified uMARS(11) |
| Stoyanov, et al(11) | 2016 | Quality | uMARS(11) |
| Stoyanov, et al(4) | 2015 | Quality | MARS(4) |
| Strandell-Laine, et al(273) | 2019 | Usability | SUS(6) |
| Stütz, et al(274) | 2017 | Usability, Acceptance | SUS(6), USE(51), modified TAM(275) |
| Symsack, et al(276) | 2020 | Usability | Modified SUS(6) |
| Talwar, et al(277) | 2019 | Quality | MARS(4) |
| Tan, et al(278) | 2019 | Usability | MARS(4) |
| Teo, et al(279) | 2019 | Usability | SUS(6) |
| Terhorst, et al(280) | 2021 | Quality | MARS(4) |
| Tinschert, et al(281) | 2017 | Quality | MARS(4) |
| Tonheim, et al(282) | 2018 | Usability | SUS(6) |
| Torbjørnsen, et al(283) | 2018 | Acceptance | The Service User Technology Acceptability Questionnaire (SUTAQ)(284) |
| Ureña, et al(285) | 2020 | Usability | SUS(6) |
| Van Cleave, et al(286) | 2019 | Acceptance | The Perceived Ease of Use and Usefulness Questionnaire(25), PSSUQ(16) |
| Vélez, et al(287) | 2014 | Usability | Health-ITUES(94) |
| Virani, et al(288) | 2019 | Quality | MARS(4) |
| Wang, et al(289) | 2018 | Usability | ASQ(37), modified PSSUQ(16) |
| Wang, et al(290) | 2021 | Quality | MARS(4) |
| Wang, et al(291) | 2020 | Quality | MARS(4) |
| Ware, et al(292) | 2019 | Satisfaction | Modified UTAUT 2(293) |
| Weekly, et al(294) | 2018 | Quality | MARS(4) |
| White, et al(295) | 2016 | Quality | MARS(4) |
| Wilson, et al(296) | 2016 | Quality | MARS(4) |
| Winoker, et al(297) | 2021 | Quality | MARS(4) |
| Woods, et al(298) | 2019 | Quality | MARS(4) |
| Woodworth, et al(299) | 2021 | Usability | SUS(6) |
| Ybarra, et al(300) | 2019 | Acceptance | Questionnaire created for a text messaging-based smoking cessation program for young adults(301) |
| Yoo, et al(302) | 2019 | Satisfaction | SUS(6) |
| Zaror, et al(303) | 2019 | Usability, Satisfaction | SUS(6), Single Ease Question (SEQ)(304) |
| Zeleke, et al(305) | 2019 | Usability | SUS(6) |
| Zhong, et al(306) | 2020 | Usability, Acceptance | SUS(6), The Questionnaire to Measure Acceptance(307) |
| Zhou, et al(271) | 2019 | Usability | MAUQ(271) |
| Zhou, et al(308) | 2019 | Usability | PSSUQ(16) |
| Zijp, et al(309) | 2020 | Acceptance | SUS(6) |

1. Alanzi T, Istepanian R, Philip N. Design and Usability Evaluation of Social Mobile Diabetes Management System in the Gulf Region. JMIR research protocols. 2016;5(3):e93.

2. Chin JP, Diehl VA, Norman KL, editors. Development of an instrument measuring user satisfaction of the human-computer interface. Proceedings of the SIGCHI conference on Human factors in computing systems; 1988.

3. Alhuwail D, Albaj R, Ahmad F, Aldakheel K. The state of mental digi-therapeutics: A systematic assessment of depression and anxiety apps available for Arabic speakers. International journal of medical informatics. 2020;135:104056.

4. Stoyanov SR, Hides L, Kavanagh DJ, Zelenko O, Tjondronegoro D, Mani M. Mobile app rating scale: a new tool for assessing the quality of health mobile apps. JMIR mHealth and uHealth. 2015;3(1):e27.

5. Alnasser A, Kyle J, Alkhalifah A, Marais D. Relationship Between Evidence Requirements, User Expectations, and Actual Experiences: Usability Evaluation of the Twazon Arabic Weight Loss App. JMIR human factors. 2018;5(2):e16.

6. Brooke J. Sus: a “quick and dirty’usability. J Usability evaluation in industry. 1996;189:189-94.

7. Alshathri DM, Alhumaimeedy AS, Al-Hudhud G, Alsaleh A, Al-Musharaf S, Aljuraiban GS. Weight Management Apps in Saudi Arabia: Evaluation of Features and Quality. JMIR mHealth and uHealth. 2020;8(10):e19844.

8. Amor-García M, Collado-Borrell R, Escudero-Vilaplana V, Melgarejo-Ortuño A, Herranz-Alonso A, Arranz Arija J, et al. Assessing Apps for Patients with Genitourinary Tumors Using the Mobile Application Rating Scale (MARS): Systematic Search in App Stores and Content Analysis. JMIR mHealth and uHealth. 2020;8(7):e17609.

9. Appleton KM, Bray J, Price S, Liebchen G, Jiang N, Mavridis I, et al. A Mobile Phone App for the Provision of Personalized Food-Based Information in an Eating-Out Situation: Development and Initial Evaluation. JMIR formative research. 2019;3(4):e12966.

10. Argent R, Slevin P, Bevilacqua A, Neligan M, Daly A, Caulfield B. Wearable Sensor-Based Exercise Biofeedback for Orthopaedic Rehabilitation: A Mixed Methods User Evaluation of a Prototype System. Sensors (Basel, Switzerland). 2019;19(2).

11. Stoyanov SR, Hides L, Kavanagh DJ, Wilson H. Development and Validation of the User Version of the Mobile Application Rating Scale (uMARS). JMIR mHealth and uHealth. 2016;4(2):e72.

12. Athilingam P, Jenkins B. Mobile Phone Apps to Support Heart Failure Self-Care Management: Integrative Review. JMIR cardio. 2018;2(1):e10057.

13. Bahadori S, Wainwright TW, Ahmed OH. Smartphone apps for total hip replacement and total knee replacement surgery patients: a systematic review. Disability and rehabilitation. 2020;42(7):983-8.

14. Bailon C, Damas M, Pomares H, Sanabria D, Perakakis P, Goicoechea C, et al. Smartphone-Based Platform for Affect Monitoring through Flexibly Managed Experience Sampling Methods. Sensors (Basel, Switzerland). 2019;19(15).

15. Bakogiannis C, Tsarouchas A, Mouselimis D, Lazaridis C, Theofillogianakos EK, Billis A, et al. A Patient-Oriented App (ThessHF) to Improve Self-Care Quality in Heart Failure: From Evidence-Based Design to Pilot Study. JMIR mHealth and uHealth. 2021;9(4):e24271.

16. Lewis JR, editor Psychometric evaluation of the post-study system usability questionnaire: The PSSUQ. Proceedings of the Human Factors Society Annual Meeting; 1992: Sage Publications Sage CA: Los Angeles, CA.

17. Balsa J, Félix I, Cláudio AP, Carmo MB, Silva ICE, Guerreiro A, et al. Usability of an Intelligent Virtual Assistant for Promoting Behavior Change and Self-Care in Older People with Type 2 Diabetes. Journal of medical systems. 2020;44(7):130.

18. Martins AI, Rosa AF, Queirós A, Silva A, Rocha NPJPCS. European portuguese validation of the system usability scale (SUS). 2015;67:293-300.

19. Baltaxe E, Embid C, Aumatell E, Martínez M, Barberan-Garcia A, Kelly J, et al. Integrated Care Intervention Supported by a Mobile Health Tool for Patients Using Noninvasive Ventilation at Home: Randomized Controlled Trial. JMIR mHealth and uHealth. 2020;8(4):e16395.

20. Reichheld FF. The one number you need to grow. Harvard business review. 2003;81(12):46-54, 124.

21. Uijen AA, Schellevis FG, van den Bosch WJ, Mokkink HG, Van Weel C, Schers HJJJoce. Nijmegen Continuity Questionnaire: development and testing of a questionnaire that measures continuity of care. 2011;64(12):1391-9.

22. Leijten FR, Hoedemakers M, Struckmann V, Kraus M, Cheraghi-Sohi S, Zemplényi A, et al. Defining good health and care from the perspective of persons with multimorbidity: results from a qualitative study of focus groups in eight European countries. 2018;8(8):e021072.

23. Bardus M, Awada N, Ghandour LA, Fares EJ, Gherbal T, Al-Zanati T, et al. The Arabic Version of the Mobile App Rating Scale: Development and Validation Study. JMIR mHealth and uHealth. 2020;8(3):e16956.

24. Barteit S, Neuhann F, Bärnighausen T, Bowa A, Wolter S, Siabwanta H, et al. Technology Acceptance and Information System Success of a Mobile Electronic Platform for Nonphysician Clinical Students in Zambia: Prospective, Nonrandomized Intervention Study. Journal of medical Internet research. 2019;21(10):e14748.

25. Davis FD. Perceived usefulness, perceived ease of use, and user acceptance of information technology. J MIS quarterly. 1989:319-40.

26. William HD, Ephraim RM. The DeLone and McLean Model of Information Systems Success: A Ten-Year Update. Journal of Management Information Systems. 2003;19(4):9-30.

27. Beatty AL, Magnusson SL, Fortney JC, Sayre GG, Whooley MA. VA FitHeart, a Mobile App for Cardiac Rehabilitation: Usability Study. JMIR human factors. 2018;5(1):e3.

28. Belarmino A, Walsh R, Alshak M, Patel N, Wu R, Hu JC. Feasibility of a Mobile Health Application To Monitor Recovery and Patient-reported Outcomes after Robot-assisted Radical Prostatectomy. European urology oncology. 2019;2(4):425-8.

29. Symer MM, Abelson JS, Milsom J, McClure B, Yeo HL. A Mobile Health Application to Track Patients After Gastrointestinal Surgery: Results from a Pilot Study. Journal of gastrointestinal surgery : official journal of the Society for Surgery of the Alimentary Tract. 2017;21(9):1500-5.

30. Bente BE, van 't Klooster J, Schreijer MA, Berkemeier L, van Gend JE, Slijkhuis PJH, et al. The Dutch COVID-19 Contact Tracing App (the CoronaMelder): Usability Study. JMIR formative research. 2021;5(3):e27882.

31. Hinderks A, Schrepp M, Domínguez Mayo FJ, Escalona MJ, Thomaschewski J. Developing a UX KPI based on the User Experience Questionnaire. Computer Standards & Interfaces. 2019;65.

32. Bentley CL, Powell L, Potter S, Parker J, Mountain GA, Bartlett YK, et al. The Use of a Smartphone App and an Activity Tracker to Promote Physical Activity in the Management of Chronic Obstructive Pulmonary Disease: Randomized Controlled Feasibility Study. JMIR mHealth and uHealth. 2020;8(6):e16203.

33. Biello KB, Hill-Rorie J, Valente PK, Futterman D, Sullivan PS, Hightow-Weidman L, et al. Development and Evaluation of a Mobile App Designed to Increase HIV Testing and Pre-exposure Prophylaxis Use Among Young Men Who Have Sex With Men in the United States: Open Pilot Trial. Journal of medical Internet research. 2021;23(3):e25107.

34. Boyle JA, Xu R, Gilbert E, Kuczynska-Burggraf M, Tan B, Teede H, et al. Ask PCOS: Identifying Need to Inform Evidence-Based App Development for Polycystic Ovary Syndrome. Seminars in reproductive medicine. 2018;36(1):59-65.

35. Brinkel J, May J, Krumkamp R, Lamshöft M, Kreuels B, Owusu-Dabo E, et al. Mobile phone-based interactive voice response as a tool for improving access to healthcare in remote areas in Ghana - an evaluation of user experiences. Tropical medicine & international health : TM & IH. 2017;22(5):622-30.

36. Broekhuis M, van Velsen L, Hermens H. Assessing usability of eHealth technology: A comparison of usability benchmarking instruments. International journal of medical informatics. 2019;128:24-31.

37. Lewis J. Psychometric evaluation of an after-scenario questionnaire for computer usability studies: The ASQ. SIGCHI Bull. 1991;23:78–81.

38. Brown HM, Bucher T, Collins CE, Rollo ME. A review of pregnancy iPhone apps assessing their quality, inclusion of behaviour change techniques, and nutrition information. Maternal & child nutrition. 2019;15(3):e12768.

39. Cervera Peris M, Alonso Rorís VM, Santos Gago JM, Álvarez Sabucedo L, Wanden-Berghe C, Sanz-Valero J. Management of the General Process of Parenteral Nutrition Using mHealth Technologies: Evaluation and Validation Study. JMIR mHealth and uHealth. 2018;6(4):e79.

40. Chang WJ, Lo SY, Kuo CL, Wang YL, Hsiao HC. Development of an intervention tool for precision oral self-care: Personalized and evidence-based practice for patients with periodontal disease. PloS one. 2019;14(11):e0225453.

41. Chapman C, Champion KE, Birrell L, Deen H, Brierley ME, Stapinski LA, et al. Smartphone Apps About Crystal Methamphetamine ("Ice"): Systematic Search in App Stores and Assessment of Composition and Quality. JMIR mHealth and uHealth. 2018;6(11):e10442.

42. Chávez A, Borrego G, Gutierrez-Garcia JO, Rodríguez LF. Design and evaluation of a mobile application for monitoring patients with Alzheimer's disease: A day center case study. International journal of medical informatics. 2019;131:103972.

43. Chen R, Santo K, Wong G, Sohn W, Spallek H, Chow C, et al. Mobile Apps for Dental Caries Prevention: Systematic Search and Quality Evaluation. JMIR mHealth and uHealth. 2021;9(1):e19958.

44. Chen YS, Wong JE, Ayob AF, Othman NE, Poh BK. Can Malaysian Young Adults Report Dietary Intake Using a Food Diary Mobile Application? A Pilot Study on Acceptability and Compliance. Nutrients. 2017;9(1).

45. Cheng H, Tutt A, Llewellyn C, Size D, Jones J, Taki S, et al. Content and Quality of Infant Feeding Smartphone Apps: Five-Year Update on a Systematic Search and Evaluation. JMIR mHealth and uHealth. 2020;8(5):e17300.

46. Cho H, Powell D, Pichon A, Thai J, Bruce J, Kuhns LM, et al. A Mobile Health Intervention for HIV Prevention Among Racially and Ethnically Diverse Young Men: Usability Evaluation. JMIR mHealth and uHealth. 2018;6(9):e11450.

47. Choi J, Baker E, Nalawade S, Lee H. Steps to Develop a Mobile App for Pain Assessment of Cancer Patients: A Usability Study. Computers, informatics, nursing : CIN. 2020;38(2):80-7.

48. Choi J, Chung C, Woo H. Diet-Related Mobile Apps to Promote Healthy Eating and Proper Nutrition: A Content Analysis and Quality Assessment. International journal of environmental research and public health. 2021;18(7).

49. Choi YK, Demiris G, Lin SY, Iribarren SJ, Landis CA, Thompson HJ, et al. Smartphone Applications to Support Sleep Self-Management: Review and Evaluation. Journal of clinical sleep medicine : JCSM : official publication of the American Academy of Sleep Medicine. 2018;14(10):1783-90.

50. Chung K, Cho HY, Park JY. A Chatbot for Perinatal Women's and Partners' Obstetric and Mental Health Care: Development and Usability Evaluation Study. JMIR medical informatics. 2021;9(3):e18607.

51. Lund A. Measuring Usability with the USE Questionnaire. Usability and User Experience Newsletter of the STC Usability SIG. 2001;8.

52. Clebone A, Strupp KM, Whitney G, Anderson MR, Hottle J, Fehr J, et al. Development and Usability Testing of the Society for Pediatric Anesthesia Pedi Crisis Mobile Application. Anesthesia and analgesia. 2019;129(6):1635-44.

53. Corazza F, Snijders D, Arpone M, Stritoni V, Martinolli F, Daverio M, et al. Development and Usability of a Novel Interactive Tablet App (PediAppRREST) to Support the Management of Pediatric Cardiac Arrest: Pilot High-Fidelity Simulation-Based Study. JMIR mHealth and uHealth. 2020;8(10):e19070.

54. Laugwitz B, Held T, Schrepp M, editors. Construction and Evaluation of a User Experience Questionnaire. HCI and Usability for Education and Work; 2008 2008//; Berlin, Heidelberg: Springer Berlin Heidelberg.

55. Cordova D, Alers-Rojas F, Lua FM, Bauermeister J, Nurenberg R, Ovadje L, et al. The Usability and Acceptability of an Adolescent mHealth HIV/STI and Drug Abuse Preventive Intervention in Primary Care. Behavioral medicine (Washington, DC). 2018;44(1):36-47.

56. Harper GW, Contreras R, Bangi A, Pedraza A. Collaborative Process Evaluation. Journal of Prevention & Intervention in the Community. 2003;26(2):53-69.

57. Larsen DL, Attkisson CC, Hargreaves WA, Nguyen TDJE, planning p. Assessment of client/patient satisfaction: development of a general scale. 1979;2(3):197-207.

58. Costa Stutzel M, Filippo MP, Sztajnberg A, da Costa R, Brites ADS, da Motta LB, et al. Multi-part quality evaluation of a customized mobile application for monitoring elderly patients with functional loss and helping caregivers. BMC medical informatics and decision making. 2019;19(1):140.

59. Messick SJ. Metric Properties of the Semantic Differential. 1957;17(2):200-6.

60. Davalbhakta S, Advani S, Kumar S, Agarwal V, Bhoyar S, Fedirko E, et al. A Systematic Review of Smartphone Applications Available for Corona Virus Disease 2019 (COVID19) and the Assessment of their Quality Using the Mobile Application Rating Scale (MARS). Journal of medical systems. 2020;44(9):164.

61. Davis A, Ellis R. A quasi-experimental investigation of college students' ratings of two physical activity mobile apps with varied behavior change technique quantity. Digital health. 2019;5:2055207619891347.

62. Day FC, Pourhomayoun M, Keeves D, Lees AF, Sarrafzadeh M, Bell D, et al. Feasibility study of an EHR-integrated mobile shared decision making application. International journal of medical informatics. 2019;124:24-30.

63. de Batlle J, Massip M, Vargiu E, Nadal N, Fuentes A, Ortega Bravo M, et al. Implementing Mobile Health-Enabled Integrated Care for Complex Chronic Patients: Patients and Professionals' Acceptability Study. JMIR mHealth and uHealth. 2020;8(11):e22136.

64. De Cock N, Van Lippevelde W, Vangeel J, Notebaert M, Beullens K, Eggermont S, et al. Feasibility and impact study of a reward-based mobile application to improve adolescents' snacking habits. Public health nutrition. 2018;21(12):2329-44.

65. Poels KdK, Y.A.W. IJsselsteijn, W.A. D3.3 : Game Experience Questionnaire. Eindhoven: Technische Universiteit Eindhoven; 2007.

66. de Dicastillo EL, García-Zapirain B, Fernández MTA, de la Torre Díez I, Oleagordia I, Celaya AA. Development and Evaluation of a Telematics Platform for Monitoring of Patients in Ambulatory Major Surgery. Telemedicine journal and e-health : the official journal of the American Telemedicine Association. 2019;25(2):152-9.

67. Deady M, Glozier N, Collins D, Einboden R, Lavender I, Wray A, et al. The Utility of a Mental Health App in Apprentice Workers: A Pilot Study. Frontiers in public health. 2020;8:389.

68. Deady M, Johnston D, Milne D, Glozier N, Peters D, Calvo R, et al. Preliminary Effectiveness of a Smartphone App to Reduce Depressive Symptoms in the Workplace: Feasibility and Acceptability Study. JMIR mHealth and uHealth. 2018;6(12):e11661.

69. Ben-Zeev D, Brenner CJ, Begale M, Duffecy J, Mohr DC, Mueser KT. Feasibility, Acceptability, and Preliminary Efficacy of a Smartphone Intervention for Schizophrenia. Schizophrenia bulletin. 2014;40(6):1244-53.

70. Devan H, Farmery D, Peebles L, Grainger R. Evaluation of Self-Management Support Functions in Apps for People With Persistent Pain: Systematic Review. JMIR mHealth and uHealth. 2019;7(2):e13080.

71. Díaz JL, Codern-Bové N, Zomeño MD, Lassale C, Schröder H, Grau M. Quantitative and qualitative evaluation of the COMPASS mobile app: a citizen science project. Informatics for health & social care. 2021:1-13.

72. Dittrich F, Back DA, Harren AK, Jäger M, Landgraeber S, Reinecke F, et al. A Possible Mobile Health Solution in Orthopedics and Trauma Surgery: Development Protocol and User Evaluation of the Ankle Joint App. JMIR mHealth and uHealth. 2020;8(2):e16403.

73. Messner E-M, Terhorst Y, Barke A, Baumeister H, Stoyanov S, Hides L, et al. The German Version of the Mobile App Rating Scale (MARS-G): Development and Validation Study. JMIR mHealth and uHealth. 2020;8(3):e14479.

74. Dunsmuir D, Wu H, Sun T, West NC, Lauder GR, Görges M, et al. A Postoperative Pain Management Mobile App (Panda) for Children at Home After Discharge: Usability and Feasibility. JMIR perioperative medicine. 2019;2(2):e12305.

75. Lewis JR. IBM computer usability satisfaction questionnaires: Psychometric evaluation and instructions for use. International Journal of Human–Computer Interaction. 1995;7(1):57-78.

76. Eapen BR, Chapman B. Mobile Access to ClinicalConnect: A User Feedback Survey on Usability, Productivity, and Quality. JMIR mHealth and uHealth. 2015;3(2):e35.

77. Ehrler F, Ducloux P, Wu DTY, Lovis C, Blondon K. Acceptance of a Mobile Application Supporting Nurses Workflow at Patient Bedside: Results from a Pilot Study. Studies in health technology and informatics. 2018;247:506-10.

78. Venkatesh V, Morris MG, Davis GB, Davis FD. User acceptance of information technology: Toward a unified view. MIS quarterly. 2003:425-78.

79. English LL, Dunsmuir D, Kumbakumba E, Ansermino JM, Larson CP, Lester R, et al. The PAediatric Risk Assessment (PARA) Mobile App to Reduce Postdischarge Child Mortality: Design, Usability, and Feasibility for Health Care Workers in Uganda. JMIR mHealth and uHealth. 2016;4(1):e16.

80. Escriche-Escuder A, De-Torres I, Roldán-Jiménez C, Martín-Martín J, Muro-Culebras A, González-Sánchez M, et al. Assessment of the Quality of Mobile Applications (Apps) for Management of Low Back Pain Using the Mobile App Rating Scale (MARS). International journal of environmental research and public health. 2020;17(24).

81. Farzandipour M, Nabovati E, Heidarzadeh Arani M, Akbari H, Sharif R, Anvari S. Enhancing Asthma Patients' Self-Management through Smartphone-Based Application: Design, Usability Evaluation, and Educational Intervention. Applied clinical informatics. 2019;10(5):870-8.

82. Fazzino TL, Martin CK, Forbush K. The Remote Food Photography Method and SmartIntake App for the Assessment of Alcohol Use in Young Adults: Feasibility Study and Comparison to Standard Assessment Methodology. JMIR mHealth and uHealth. 2018;6(9):e10460.

83. Ferguson MA, Maidment DW, Gomez R, Coulson N, Wharrad H. The feasibility of an m-health educational programme (m2Hear) to improve outcomes in first-time hearing aid users. International journal of audiology. 2020:1-12.

84. Fijačko N, Gosak L, Cilar L, Novšak A, Creber RM, Skok P, et al. The Effects of Gamification and Oral Self-Care on Oral Hygiene in Children: Systematic Search in App Stores and Evaluation of Apps. JMIR mHealth and uHealth. 2020;8(7):e16365.

85. Floryan M, Chow PI, Schueller SM, Ritterband LM. The Model of Gamification Principles for Digital Health Interventions: Evaluation of Validity and Potential Utility. Journal of medical Internet research. 2020;22(6):e16506.

86. Fraynt R, Cooper D, Edwards-Stewart A, Hoyt T, Micheel L, Pruitt L, et al. An evaluation of mobile applications designed to assist service members and veterans transitioning to civilian life. Psychological services. 2018;15(2):208-15.

87. Fu MR, Axelrod D, Guth AA, Rampertaap K, El-Shammaa N, Hiotis K, et al. mHealth self-care interventions: managing symptoms following breast cancer treatment. mHealth. 2016;2.

88. Fuller-Tyszkiewicz M, Richardson B, Klein B, Skouteris H, Christensen H, Austin D, et al. A Mobile App-Based Intervention for Depression: End-User and Expert Usability Testing Study. JMIR mental health. 2018;5(3):e54.

89. Furlong L, Morris M, Serry T, Erickson S. Mobile apps for treatment of speech disorders in children: An evidence-based analysis of quality and efficacy. PloS one. 2018;13(8):e0201513.

90. Galve Villa M, T SP, Cid Royo A, C RB, Boudreau SA. Digital Pain Mapping and Tracking in Patients With Chronic Pain: Longitudinal Study. Journal of medical Internet research. 2020;22(10):e21475.

91. Gamwell KL, Kollin SR, Gibler RC, Bedree H, Bieniak KH, Jagpal A, et al. Systematic evaluation of commercially available pain management apps examining behavior change techniques. Pain. 2021;162(3):856-65.

92. Gandhi S, Morillo CA, Schwalm JD. Implantable Cardioverter Defibrillator mHealth App for Physician Referrals and eHealth Education: ICD-TEACH Pilot Study. JMIR cardio. 2018;2(2):e10499.

93. Gannon B, Davis R, Kuhns LM, Rodriguez RG, Garofalo R, Schnall R. A Mobile Sexual Health App on Empowerment, Education, and Prevention for Young Adult Men (MyPEEPS Mobile): Acceptability and Usability Evaluation. JMIR formative research. 2020;4(4):e17901.

94. Yen P-Y, Wantland D, Bakken S. Development of a Customizable Health IT Usability Evaluation Scale. AMIA Annual Symposium proceedings / AMIA Symposium AMIA Symposium. 2010;2010:917-21.

95. Garzo A, Silva PA, Garay-Vitoria N, Hernandez E, Cullen S, Cochen De Cock V, et al. Design and development of a gait training system for Parkinson's disease. PloS one. 2018;13(11):e0207136.

96. Georgsson M, Staggers N. An evaluation of patients' experienced usability of a diabetes mHealth system using a multi-method approach. Journal of biomedical informatics. 2016;59:115-29.

97. Maguire M. Methods to Support Human-Centred Design. International Journal of Human-Computer Studies. 2001;55:587-634.

98. Georgsson M, Staggers N. Quantifying usability: an evaluation of a diabetes mHealth system on effectiveness, efficiency, and satisfaction metrics with associated user characteristics. Journal of the American Medical Informatics Association : JAMIA. 2016;23(1):5-11.

99. Ghanbari H, Ansari S, Ghannam M, Lathkar-Pradhan S, Kratz A, Oral H, et al. Feasibility and Usability of a Mobile Application to Assess Symptoms and Affect in Patients with Atrial Fibrillation: A Pilot Study. Journal of atrial fibrillation. 2017;10(2):1672.

100. Bhattacharyya MR, Steptoe A. Emotional triggers of acute coronary syndromes: strength of evidence, biological processes, and clinical implications. Progress in cardiovascular diseases. 2007;49(5):353-65.

101. Gill RK, Ogilvie G, Norman WV, Fitzsimmons B, Maher C, Renner R. Feasibility and Acceptability of a Mobile Technology Intervention to Support Postabortion Care (The FACTS Study Phase II) After Surgical Abortion: User-Centered Design. JMIR human factors. 2019;6(4):e14558.

102. Gong E, Zhang Z, Jin X, Liu Y, Zhong L, Wu Y, et al. Quality, Functionality, and Features of Chinese Mobile Apps for Diabetes Self-Management: Systematic Search and Evaluation of Mobile Apps. JMIR mHealth and uHealth. 2020;8(4):e14836.

103. Grainger R, Townsley H, White B, Langlotz T, Taylor WJ. Apps for People With Rheumatoid Arthritis to Monitor Their Disease Activity: A Review of Apps for Best Practice and Quality. JMIR mHealth and uHealth. 2017;5(2):e7.

104. Grainger R, Townsley HR, Ferguson CA, Riley FE, Langlotz T, Taylor WJ. Patient and clinician views on an app for rheumatoid arthritis disease monitoring: Function, implementation and implications. International journal of rheumatic diseases. 2020;23(6):813-27.

105. Grasaas E, Fegran L, Helseth S, Stinson J, Martinez S, Lalloo C, et al. iCanCope With Pain: Cultural Adaptation and Usability Testing of a Self-Management App for Adolescents With Persistent Pain in Norway. JMIR research protocols. 2019;8(6):e12940.

106. Grindrod KA, Gates A, Dolovich L, Slavcev R, Drimmie R, Aghaei B, et al. ClereMed: Lessons Learned From a Pilot Study of a Mobile Screening Tool to Identify and Support Adults Who Have Difficulty With Medication Labels. JMIR mHealth and uHealth. 2014;2(3):e35.

107. Ha YP, Tesfalul MA, Littman-Quinn R, Antwi C, Green RS, Mapila TO, et al. Evaluation of a Mobile Health Approach to Tuberculosis Contact Tracing in Botswana. Journal of health communication. 2016;21(10):1115-21.

108. Hafiz P, Bardram JE. The Ubiquitous Cognitive Assessment Tool for Smartwatches: Design, Implementation, and Evaluation Study. JMIR mHealth and uHealth. 2020;8(6):e17506.

109. Hemmings NR, Kawadler JM, Whatmough R, Ponzo S, Rossi A, Morelli D, et al. Development and Feasibility of a Digital Acceptance and Commitment Therapy-Based Intervention for Generalized Anxiety Disorder: Pilot Acceptability Study. JMIR formative research. 2021;5(2):e21737.

110. Herbuela V, Karita T, Francisco ME, Watanabe K. An Integrated mHealth App for Dengue Reporting and Mapping, Health Communication, and Behavior Modification: Development and Assessment of Mozzify. JMIR formative research. 2020;4(1):e16424.

111. Hill JR, Harrington AB, Adeoye P, Campbell NL, Holden RJ. Going Remote-Demonstration and Evaluation of Remote Technology Delivery and Usability Assessment With Older Adults: Survey Study. JMIR mHealth and uHealth. 2021;9(3):e26702.

112. Holden RJ. A Simplified System Usability Scale (SUS) for Cognitively Impaired and Older Adults. 2020;9(1):180-2.

113. Ho L, Macnab A, Matsubara Y, Peterson K, Tsang B, Stothers L. Rating of Pelvic Floor Muscle Training Mobile Applications for Treatment of Urinary Incontinence in Women. Urology. 2021;150:92-8.

114. Hoffmann A, Faust-Christmann CA, Zolynski G, Bleser G. Toward Gamified Pain Management Apps: Mobile Application Rating Scale-Based Quality Assessment of Pain-Mentor's First Prototype Through an Expert Study. JMIR formative research. 2020;4(5):e13170.

115. Holden RJ, Campbell NL, Abebe E, Clark DO, Ferguson D, Bodke K, et al. Usability and feasibility of consumer-facing technology to reduce unsafe medication use by older adults. Research in social & administrative pharmacy : RSAP. 2020;16(1):54-61.

116. Hsieh KL, Fanning JT, Rogers WA, Wood TA, Sosnoff JJ. A Fall Risk mHealth App for Older Adults: Development and Usability Study. JMIR aging. 2018;1(2):e11569.

117. Hyatt A, Lipson-Smith R, Morkunas B, Krishnasamy M, Jefford M, Baxter K, et al. Testing Consultation Recordings in a Clinical Setting With the SecondEars Smartphone App: Mixed Methods Implementation Study. JMIR mHealth and uHealth. 2020;8(1):e15593.

118. Islam MN, Karim MM, Inan TT, Islam A. Investigating usability of mobile health applications in Bangladesh. BMC medical informatics and decision making. 2020;20(1):19.

119. Ithnin M, Mohd Rani MD, Abd Latif Z, Kani P, Syaiful A, Nor Aripin KN, et al. Mobile App Design, Development, and Publication for Adverse Drug Reaction Assessments of Causality, Severity, and Preventability. JMIR mHealth and uHealth. 2017;5(5):e78.

120. Jacobson AE, Vesely SK, Haamid F, Christian-Rancy M, O'Brien SH. Mobile Application vs Paper Pictorial Blood Assessment Chart to Track Menses in Young Women: A Randomized Cross-over Design. Journal of pediatric and adolescent gynecology. 2018;31(2):84-8.

121. Lim MSC, Sacks-Davis R, Aitken CK, Hocking JS, Hellard ME. Randomised controlled trial of paper, online and SMS diaries for collecting sexual behaviour information from young people. 2010;64(10):885-9.

122. Jain YS, Garg A, Jhamb DK, Jain P, Karar A. Preparing India to Leverage Power of Mobile Technology: Development of a Bilingual Mobile Health Tool for Heart Patients. Cardiovascular & hematological agents in medicinal chemistry. 2019;17(2):125-34.

123. Cho MJ, Sim JL, Hwang SY. Development of smartphone educational application for patients with coronary artery disease. Healthcare informatics research. 2014;20(2):117-24.

124. Jeon E, Park HA. Development of a smartphone application for clinical-guideline-based obesity management. Healthcare informatics research. 2015;21(1):10-20.

125. Ji M, Wu Y, Chang P, Yang X, Yang F, Xu S. Development and Usability Evaluation of the Mobile Delirium Assessment App Based on Confusion Assessment Method for Intensive Care Unit (CAM-ICU). Studies in health technology and informatics. 2015;216:899.

126. Ji Y, Plourde H, Bouzo V, Kilgour RD, Cohen TR. Validity and Usability of a Smartphone Image-Based Dietary Assessment App Compared to 3-Day Food Diaries in Assessing Dietary Intake Among Canadian Adults: Randomized Controlled Trial. JMIR mHealth and uHealth. 2020;8(9):e16953.

127. Jones C, O'Toole K, Jones K, Brémault-Phillips S. Quality of Psychoeducational Apps for Military Members With Mild Traumatic Brain Injury: An Evaluation Utilizing the Mobile Application Rating Scale. JMIR mHealth and uHealth. 2020;8(8):e19807.

128. Jonker LT, Plas M, de Bock GH, Buskens E, van Leeuwen BL, Lahr MMH. Remote Home Monitoring of Older Surgical Cancer Patients: Perspective on Study Implementation and Feasibility. Annals of surgical oncology. 2021;28(1):67-78.

129. Jovičić S, Siodmiak J, Alcorta MD, Kittel M, Oosterhuis W, Aakre KM, et al. Quality benchmarking of smartphone laboratory medicine applications: comparison of laboratory medicine specialists' and non-laboratory medicine professionals' evaluation. Clinical chemistry and laboratory medicine. 2020.

130. Jovičić S, Siodmiak J, Watson ID. Quality evaluation of smartphone applications for laboratory medicine. Clinical chemistry and laboratory medicine. 2019;57(3):388-97.

131. Juengst SB, Graham KM, Pulantara IW, McCue M, Whyte EM, Dicianno BE, et al. Pilot feasibility of an mHealth system for conducting ecological momentary assessment of mood-related symptoms following traumatic brain injury. Brain injury. 2015;29(11):1351-61.

132. Parmanto B, Lewis AN, Jr., Graham KM, Bertolet MH. Development of the Telehealth Usability Questionnaire (TUQ). Int J Telerehabil. 2016;8(1):3-10.

133. Jung H, Demiris G, Tarczy-Hornoch P, Zachry M. A Novel Food Record App for Dietary Assessments Among Older Adults With Type 2 Diabetes: Development and Usability Study. JMIR formative research. 2021;5(2):e14760.

134. Jupp JCY, Sultani H, Cooper CA, Peterson KA, Truong TH. Evaluation of mobile phone applications to support medication adherence and symptom management in oncology patients. Pediatric blood & cancer. 2018;65(11):e27278.

135. Kalhori SRN, Hemmat M, Noori T, Heydarian S, Katigari MR. Quality Evaluation of English Mobile Applications for Gestational Diabetes: App Review using Mobile Application Rating Scale (MARS). Current diabetes reviews. 2021;17(2):161-8.

136. Kalz M, Lenssen N, Felzen M, Rossaint R, Tabuenca B, Specht M, et al. Smartphone apps for cardiopulmonary resuscitation training and real incident support: a mixed-methods evaluation study. Journal of medical Internet research. 2014;16(3):e89.

137. Khowaja K, Al-Thani D. New Checklist for the Heuristic Evaluation of mHealth Apps (HE4EH): Development and Usability Study. JMIR mHealth and uHealth. 2020;8(10):e20353.

138. Kim MS, Aro MR, Lage KJ, Ingalls KL, Sindhwani V, Markey MK. Exploring the Usability of Mobile Apps Supporting Radiologists' Training in Diagnostic Decision Making. Journal of the American College of Radiology : JACR. 2016;13(3):335-43.

139. Kim Y, Lee H, Lee MK, Lee H, Jang H. Development of a Living Lab for a Mobile-Based Health Program for Korean-Chinese Working Women in South Korea: Mixed Methods Study. JMIR mHealth and uHealth. 2020;8(1):e15359.

140. Jin M, Kim J. Development and Evaluation of an Evaluation Tool for Healthcare Smartphone Applications. Telemedicine journal and e-health : the official journal of the American Telemedicine Association. 2015;21(10):831-7.

141. Kizakevich PN, Eckhoff R, Brown J, Tueller SJ, Weimer B, Bell S, et al. PHIT for Duty, a Mobile Application for Stress Reduction, Sleep Improvement, and Alcohol Moderation. Military medicine. 2018;183(suppl_1):353-63.

142. Kizakevich PN, Eckhoff R, Weger S, Weeks A, Brown J, Bryant S, et al. A personal health information toolkit for health intervention research. Studies in health technology and informatics. 2014;199:35-9.

143. Klingberg A, Wallis LA, Hasselberg M, Yen PY, Fritzell SC. Teleconsultation Using Mobile Phones for Diagnosis and Acute Care of Burn Injuries Among Emergency Physicians: Mixed-Methods Study. JMIR mHealth and uHealth. 2018;6(10):e11076.

144. Klingemann H, Flückiger M, Bongard T, Büchi M, Carrara M. Design and Content Quality of Alcohol-Related German, French and Italian Self-Tracking Applications. Substance use & misuse. 2020;55(5):851-9.

145. Kooij L, Vos PJE, Dijkstra A, van Harten WH. Effectiveness of a Mobile Health and Self-Management App for High-Risk Patients With Chronic Obstructive Pulmonary Disease in Daily Clinical Practice: Mixed Methods Evaluation Study. JMIR mHealth and uHealth. 2021;9(2):e21977.

146. Kristjansdottir OB, Børøsund E, Westeng M, Ruland C, Stenberg U, Zangi HA, et al. Mobile App to Help People With Chronic Illness Reflect on Their Strengths: Formative Evaluation and Usability Testing. JMIR formative research. 2020;4(3):e16831.

147. Kuhns LM, Hereth J, Garofalo R, Hidalgo M, Johnson AK, Schnall R, et al. A Uniquely Targeted, Mobile App-Based HIV Prevention Intervention for Young Transgender Women: Adaptation and Usability Study. Journal of medical Internet research. 2021;23(3):e21839.

148. Kwan YH, Ong WJ, Xiong M, Leung YY, Phang JK, Wang CTM, et al. Evaluation of Mobile Apps Targeted at Patients With Spondyloarthritis for Disease Monitoring: Systematic App Search. JMIR mHealth and uHealth. 2019;7(10):e14753.

149. Lambert K, Mullan J, Mansfield K, Owen P. Should We Recommend Renal Diet-Related Apps to Our Patients? An Evaluation of the Quality and Health Literacy Demand of Renal Diet-Related Mobile Applications. Journal of renal nutrition : the official journal of the Council on Renal Nutrition of the National Kidney Foundation. 2017;27(6):430-8.

150. Silberg WM, Lundberg GD, Musacchio RA. Assessing, controlling, and assuring the quality of medical information on the Internet: Caveant lector et viewor--Let the reader and viewer beware. Jama. 1997;277(15):1244-5.

151. Langlet B, Maramis C, Diou C, Maglaveras N, Fagerberg P, Heimeier R, et al. Formative Evaluation of a Smartphone App for Monitoring Daily Meal Distribution and Food Selection in Adolescents: Acceptability and Usability Study. JMIR mHealth and uHealth. 2020;8(7):e14778.

152. Langlet BS, Odegi D, Zandian M, Nolstam J, Södersten P, Bergh C. Virtual Reality App for Treating Eating Behavior in Eating Disorders: Development and Usability Study. JMIR serious games. 2021;9(2):e24998.

153. Lawitschka A, Buehrer S, Bauer D, Peters K, Silbernagl M, Zubarovskaya N, et al. A Web-Based Mobile App (INTERACCT App) for Adolescents Undergoing Cancer and Hematopoietic Stem Cell Transplantation Aftercare to Improve the Quality of Medical Information for Clinicians: Observational Study. JMIR mHealth and uHealth. 2020;8(6):e18781.

154. LeBeau K, Huey LG, Hart M. Assessing the Quality of Mobile Apps Used by Occupational Therapists: Evaluation Using the User Version of the Mobile Application Rating Scale. JMIR mHealth and uHealth. 2019;7(5):e13019.

155. Li H, Yang S, Chi H, Xu L, Zhang T, Singleton G, et al. Enhancing attention and memory of individuals at clinical high risk for psychosis with mHealth technology. Asian journal of psychiatry. 2021;58:102587.

156. Li Y, Ding J, Wang Y, Tang C, Zhang P. Nutrition-Related Mobile Apps in the China App Store: Assessment of Functionality and Quality. JMIR mHealth and uHealth. 2019;7(7):e13261.

157. Liao J, Xiao HY, Li XQ, Sun SH, Liu SX, Yang YJ, et al. A Social Group-Based Information-Motivation-Behavior Skill Intervention to Promote Acceptability and Adoption of Wearable Activity Trackers Among Middle-Aged and Older Adults: Cluster Randomized Controlled Trial. JMIR mHealth and uHealth. 2020;8(4):e14969.

158. McMahon SK, Lewis B, Oakes M, Guan W, Wyman JF, Rothman AJ. Older Adults’ Experiences Using a Commercially Available Monitor to Self-Track Their Physical Activity. JMIR mHealth and uHealth. 2016;4(2):e35.

159. Lim J, Cloete G, Dunsmuir DT, Payne BA, Scheffer C, von Dadelszen P, et al. Usability and Feasibility of PIERS on the Move: An mHealth App for Pre-Eclampsia Triage. JMIR mHealth and uHealth. 2015;3(2):e37.

160. Lim JH, Lim CK, Ibrahim I, Syahrul J, Mohamed Zabil MH, Zakaria NF, et al. Limitations of Existing Dialysis Diet Apps in Promoting User Engagement and Patient Self-Management: Quantitative Content Analysis Study. JMIR mHealth and uHealth. 2020;8(6):e13808.

161. Liu AY, Laborde ND, Coleman K, Vittinghoff E, Gonzalez R, Wilde G, et al. DOT Diary: Developing a Novel Mobile App Using Artificial Intelligence and an Electronic Sexual Diary to Measure and Support PrEP Adherence Among Young Men Who Have Sex with Men. AIDS and behavior. 2021;25(4):1001-12.

162. Liu HT, Chia RM, Setiawan IMA, Crytzer TM, Ding D. Development of "My Wheelchair Guide" app: a qualitative study. Disability and rehabilitation Assistive technology. 2019;14(8):839-48.

163. Liu XH, Jin F, Hsu J, Li DN, Chen W. Comparing Smartphone Apps for Traditional Chinese Medicine and Modern Medicine in China: Systematic Search and Content Analysis. JMIR mHealth and uHealth. 2021;9(3):e27406.

164. Liu YC, Chen CH, Lin YS, Chen HY, Irianti D, Jen TN, et al. Design and Usability Evaluation of Mobile Voice-Added Food Reporting for Elderly People: Randomized Controlled Trial. JMIR mHealth and uHealth. 2020;8(9):e20317.

165. Liu YC, Wu ST, Lin SJ, Chen CH, Lin YS, Chen HY. Usability of Food Size Aids in Mobile Dietary Reporting Apps for Young Adults: Randomized Controlled Trial. JMIR mHealth and uHealth. 2020;8(4):e14543.

166. Logsdon MC, Lauf A, Stikes R, Revels A, Vickers-Smith R. Partnering with new mothers to develop a smart phone app to prevent maternal mortality after hospital discharge: A pilot study. Journal of advanced nursing. 2020;76(1):324-7.

167. Loh KP, Ramsdale E, Culakova E, Mendler JH, Liesveld JL, O'Dwyer KM, et al. Novel mHealth App to Deliver Geriatric Assessment-Driven Interventions for Older Adults With Cancer: Pilot Feasibility and Usability Study. JMIR cancer. 2018;4(2):e10296.

168. Robinson MD, Branham AR, Locklear A, Robertson S, Gridley T. Measuring Satisfaction and Usability of FaceTime for Virtual Visits in Patients with Uncontrolled Diabetes. Telemedicine journal and e-health : the official journal of the American Telemedicine Association. 2016;22(2):138-43.

169. Lozano-Lozano M, Cantarero-Villanueva I, Martin-Martin L, Galiano-Castillo N, Sanchez MJ, Fernández-Lao C, et al. A Mobile System to Improve Quality of Life Via Energy Balance in Breast Cancer Survivors (BENECA mHealth): Prospective Test-Retest Quasiexperimental Feasibility Study. JMIR mHealth and uHealth. 2019;7(6):e14136.

170. Lozano-Lozano M, Galiano-Castillo N, Fernández-Lao C, Postigo-Martin P, Álvarez-Salvago F, Arroyo-Morales M, et al. The Ecofisio Mobile App for Assessment and Diagnosis Using Ultrasound Imaging for Undergraduate Health Science Students: Multicenter Randomized Controlled Trial. Journal of medical Internet research. 2020;22(3):e16258.

171. Fernández-Lao C, Cantarero-Villanueva I, Galiano-Castillo N, Caro-Morán E, Díaz-Rodríguez L, Arroyo-Morales M. The effectiveness of a mobile application for the development of palpation and ultrasound imaging skills to supplement the traditional learning of physiotherapy students. BMC medical education. 2016;16(1):274.

172. Lu DJ, Girgis M, David JM, Chung EM, Atkins KM, Kamrava M. Evaluation of Mobile Health Applications to Track Patient-Reported Outcomes for Oncology Patients: A Systematic Review. Advances in radiation oncology. 2021;6(1):100576.

173. Luna-Perejon F, Malwade S, Styliadis C, Civit J, Cascado-Caballero D, Konstantinidis E, et al. Evaluation of user satisfaction and usability of a mobile app for smoking cessation. Computer methods and programs in biomedicine. 2019;182:105042.

174. Wuensch KL. What is a likert scale? And how do you pronounce ‘likert?’, What is a likert scale? And how do you pronounce ‘likert?’,. East Carolina University. 2005.

175. Korhonen H, Koivisto E. Playability heuristics for mobile games2006. 9-16 p.

176. Luo S, Botash AS. Testing a mobile app for child abuse treatment: A mixed methods study. International journal of nursing sciences. 2020;7(3):320-9.

177. Tariman JD, Berry DL, Halpenny B, Wolpin S, Schepp K. Validation and testing of the Acceptability E-scale for web-based patient-reported outcomes in cancer care. Applied nursing research : ANR. 2011;24(1):53-8.

178. Luštrek M, Bohanec M, Cavero Barca C, Ciancarelli MC, Clays E, Dawodu AA, et al. A Personal Health System for Self-Management of Congestive Heart Failure (HeartMan): Development, Technical Evaluation, and Proof-of-Concept Randomized Controlled Trial. JMIR medical informatics. 2021;9(3):e24501.

179. Lyzwinski LN, Edirippulige S, Caffery L, Bambling M. Mindful Eating Mobile Health Apps: Review and Appraisal. JMIR mental health. 2019;6(8):e12820.

180. Macedo FS, Silva PGB, Marçal de Barros Filho E, Rolim J. Evaluation of Usability, Perception of Usefulness, and Efficiency of an Application in Interpreting Imaging Examinations and Supporting Decision-Making in Orthopedics. Telemedicine journal and e-health : the official journal of the American Telemedicine Association. 2020.

181. Maguire R, Connaghan J, Arber A, Klepacz N, Blyth KG, McPhelim J, et al. Advanced Symptom Management System for Patients with Malignant Pleural Mesothelioma (ASyMSmeso): Mixed Methods Study. Journal of medical Internet research. 2020;22(11):e19180.

182. Wilson EV, Lankton NK. Modeling patients' acceptance of provider-delivered e-health. Journal of the American Medical Informatics Association : JAMIA. 2004;11(4):241-8.

183. Mandracchia F, Llauradó E, Tarro L, Valls RM, Solà R. Mobile Phone Apps for Food Allergies or Intolerances in App Stores: Systematic Search and Quality Assessment Using the Mobile App Rating Scale (MARS). JMIR mHealth and uHealth. 2020;8(9):e18339.

184. Mani M, Kavanagh DJ, Hides L, Stoyanov SR. Review and Evaluation of Mindfulness-Based iPhone Apps. JMIR mHealth and uHealth. 2015;3(3):e82.

185. Marques ADB, Moreira TMM, Jorge TV, Rabelo SMS, Carvalho R, Felipe GF. Usability of a mobile application on diabetic foot self-care. Revista brasileira de enfermagem. 2020;73(4):e20180862.

186. Wangenheim CG BA, Nunes JV, Lacerda TC, Oliveira RJ, Krone C, et al., editor Sure: uma proposta de questionário e escala para

avaliar a usabilidade de aplicações para smartphones pós-teste de usabilidade. 6ta Conferencia Latinoamericana de Diseño de

Interacción; 2014.

187. Martin Payo R, Fernandez Álvarez MM, Blanco Díaz M, Cuesta Izquierdo M, Stoyanov SR, Llaneza Suárez E. Spanish adaptation and validation of the Mobile Application Rating Scale questionnaire. International journal of medical informatics. 2019;129:95-9.

188. Martín Payo R, Harris J, Armes J. Prescribing fitness apps for people with cancer: a preliminary assessment of content and quality of commercially available apps. Journal of cancer survivorship : research and practice. 2019;13(3):397-405.

189. Masterson Creber RM, Maurer MS, Reading M, Hiraldo G, Hickey KT, Iribarren S. Review and Analysis of Existing Mobile Phone Apps to Support Heart Failure Symptom Monitoring and Self-Care Management Using the Mobile Application Rating Scale (MARS). JMIR mHealth and uHealth. 2016;4(2):e74.

190. Mattson DC. Usability evaluation of the digital anger thermometer app. Health informatics journal. 2017;23(3):234-45.

191. Gediga G, Hamborg K-C, Düntsch I. The IsoMetrics usability inventory: an operationalization of ISO 9241-10 supporting summative and formative evaluation of software systems. Behaviour & information technology. 1999;18(3):151-64.

192. Mauch CE, Wycherley TP, Laws RA, Johnson BJ, Bell LK, Golley RK. Mobile Apps to Support Healthy Family Food Provision: Systematic Assessment of Popular, Commercially Available Apps. JMIR mHealth and uHealth. 2018;6(12):e11867.

193. Mayoral K, Garin O, Caballero-Rabasco MA, Praena-Crespo M, Bercedo A, Hernandez G, et al. Smartphone App for monitoring Asthma in children and adolescents. Quality of life research : an international journal of quality of life aspects of treatment, care and rehabilitation. 2021.

194. Meedya S, Win K, Yeatman H, Fahy K, Walton K, Burgess L, et al. Developing and testing a mobile application for breastfeeding support: The Milky Way application. Women and birth : journal of the Australian College of Midwives. 2021;34(2):e196-e203.

195. Hoehle H, Venkatesh V. Mobile application usability: conceptualization and instrument development. MIS Q. 2015;39(2):435–72.

196. Mehdi M, Stach M, Riha C, Neff P, Dode A, Pryss R, et al. Smartphone and Mobile Health Apps for Tinnitus: Systematic Identification, Analysis, and Assessment. JMIR mHealth and uHealth. 2020;8(8):e21767.

197. Messner EM, Terhorst Y, Barke A, Baumeister H, Stoyanov S, Hides L, et al. The German Version of the Mobile App Rating Scale (MARS-G): Development and Validation Study. JMIR mHealth and uHealth. 2020;8(3):e14479.

198. Metelmann B, Metelmann C, Schuffert L, Hahnenkamp K, Brinkrolf P. Medical Correctness and User Friendliness of Available Apps for Cardiopulmonary Resuscitation: Systematic Search Combined With Guideline Adherence and Usability Evaluation. JMIR mHealth and uHealth. 2018;6(11):e190.

199. Moeini S, Watzlaf V, Zhou L, Abernathy RP. Development of a Weighted Well-Being Assessment Mobile App for Trauma Affected Communities: A Usability Study. Perspectives in health information management. 2021;18(Winter):1o.

200. Mohamad Marzuki MF, Yaacob NA, Bin Yaacob NM, Abu Hassan MR, Ahmad SB. Usable Mobile App for Community Education on Colorectal Cancer: Development Process and Usability Study. JMIR human factors. 2019;6(2):e12103.

201. Mohamad Marzuki MF, Yaacob NA, Yaacob NM. Translation, Cross-Cultural Adaptation, and Validation of the Malay Version of the System Usability Scale Questionnaire for the Assessment of Mobile Apps. JMIR human factors. 2018;5(2):e10308.

202. Mohseni Moallem Kolaei N, Ayatollahi H, Elyasi F. Delirium in Burn Patients: Developing a Mobile Application for Assessment and Diagnosis. Journal of burn care & research : official publication of the American Burn Association. 2021;42(1):87-92.

203. Monteiro-Guerra F, Signorelli GR, Tadas S, Dorronzoro Zubiete E, Rivera Romero O, Fernandez-Luque L, et al. A Personalized Physical Activity Coaching App for Breast Cancer Survivors: Design Process and Early Prototype Testing. JMIR mHealth and uHealth. 2020;8(7):e17552.

204. Frøkjær E, Hertzum M, Hornbæk K. Measuring usability: are effectiveness, efficiency, and satisfaction really correlated? Proceedings of the SIGCHI conference on Human Factors in Computing Systems; The Hague, The Netherlands: Association for Computing Machinery; 2000. p. 345–52.

205. Moradian S, Krzyzanowska MK, Maguire R, Morita PP, Kukreti V, Avery J, et al. Usability Evaluation of a Mobile Phone-Based System for Remote Monitoring and Management of Chemotherapy-Related Side Effects in Cancer Patients: Mixed-Methods Study. JMIR cancer. 2018;4(2):e10932.

206. Ajzen I. CONSTRUCTING A THEORY OF PLANNED BEHAVIOR QUESTIONNAIRE 2006 [Available from: <http://people.umass.edu/aizen/pdf/tpb.measurement.pdf>.

207. Moral-Munoz JA, Esteban-Moreno B, Herrera-Viedma E, Cobo MJ, Pérez IJ. Smartphone Applications to Perform Body Balance Assessment: a Standardized Review. Journal of medical systems. 2018;42(7):119.

208. Morita PP, Yeung MS, Ferrone M, Taite AK, Madeley C, Stevens Lavigne A, et al. A Patient-Centered Mobile Health System That Supports Asthma Self-Management (breathe): Design, Development, and Utilization. JMIR mHealth and uHealth. 2019;7(1):e10956.

209. Moura J, Almeida AMP, Roque F, Figueiras A, Herdeiro MT. A Mobile App to Support Clinical Diagnosis of Upper Respiratory Problems (eHealthResp): Co-Design Approach. Journal of medical Internet research. 2021;23(1):e19194.

210. D. T. 247 web usability guidelines 2016 [Available from: <https://www.userfocus.co.uk/resources/guidelines.html>.

211. Muhindo M, Bress J, Kalanda R, Armas J, Danziger E, Kamya MR, et al. Implementation of a Newborn Clinical Decision Support Software (NoviGuide) in a Rural District Hospital in Eastern Uganda: Feasibility and Acceptability Study. JMIR mHealth and uHealth. 2021;9(2):e23737.

212. Kirkpatrick DL. Techniques for evaluating training programs. Training and development journal. 1979.

213. Musgrave LM, Kizirian NV, Homer CSE, Gordon A. Mobile Phone Apps in Australia for Improving Pregnancy Outcomes: Systematic Search on App Stores. JMIR mHealth and uHealth. 2020;8(11):e22340.

214. Müssener U, Thomas K, Linderoth C, Löf M, Åsberg K, Henriksson P, et al. Development of an Intervention Targeting Multiple Health Behaviors Among High School Students: Participatory Design Study Using Heuristic Evaluation and Usability Testing. JMIR mHealth and uHealth. 2020;8(10):e17999.

215. Newton A, Bagnell A, Rosychuk R, Duguay J, Wozney L, Huguet A, et al. A Mobile Phone-Based App for Use During Cognitive Behavioral Therapy for Adolescents With Anxiety (MindClimb): User-Centered Design and Usability Study. JMIR mHealth and uHealth. 2020;8(12):e18439.

216. Nguyen AT, Somerville EK, Espín-Tello SM, Keglovits M, Stark SL. A Mobile App Directory of Occupational Therapists Who Provide Home Modifications: Development and Preliminary Usability Evaluation. JMIR rehabilitation and assistive technologies. 2020;7(1):e14465.

217. Nguyen M, Hossain N, Tangri R, Shah J, Agarwal P, Thompson-Hutchison F, et al. Systematic Evaluation of Canadian Diabetes Smartphone Applications for People With Type 1, Type 2 and Gestational Diabetes. Canadian journal of diabetes. 2021;45(2):174-8.e1.

218. Olaoye O, Tuck C, Khor WP, McMenamin R, Hudson L, Northall M, et al. Improving Access to Antimicrobial Prescribing Guidelines in 4 African Countries: Development and Pilot Implementation of an App and Cross-Sectional Assessment of Attitudes and Behaviour Survey of Healthcare Workers and Patients. Antibiotics (Basel, Switzerland). 2020;9(9).

219. Panesar P, Jones A, Aldous A, Kranzer K, Halpin E, Fifer H, et al. Attitudes and Behaviours to Antimicrobial Prescribing following Introduction of a Smartphone App. PloS one. 2016;11(4):e0154202.

220. Ong JG, Lim-Ashworth NS, Ooi YP, Boon JS, Ang RP, Goh DH, et al. An Interactive Mobile App Game to Address Aggression (RegnaTales): Pilot Quantitative Study. JMIR serious games. 2019;7(2):e13242.

221. Moser C, Fuchsberger V, Tscheligi M. Rapid assessment of game experiences in public settings. Proceedings of the 4th International Conference on Fun and Games; Toulouse, France: Association for Computing Machinery; 2012. p. 73–82.

222. Tan JL, Goh DH-L, Ang RP, Huan VS, editors. Usability and playability heuristics for evaluation of an instructional game. E-Learn: World Conference on E-Learning in Corporate, Government, Healthcare, and Higher Education; 2010: Association for the Advancement of Computing in Education (AACE).

223. Ooi YP, Goh DH-L, Mekler ED, Tuch AN, Boon J, Ang RP, et al. Understanding player perceptions of RegnaTales, a mobile game for teaching social problem solving skills. Proceedings of the 31st Annual ACM Symposium on Applied Computing; Pisa, Italy: Association for Computing Machinery; 2016. p. 167–72.

224. O'Reilly MA, Slevin P, Ward T, Caulfield B. A Wearable Sensor-Based Exercise Biofeedback System: Mixed Methods Evaluation of Formulift. JMIR mHealth and uHealth. 2018;6(1):e33.

225. Oyetunde OO, Ogidan O, Akinyemi MI, Ogunbameru AA, Asaolu OF. Mobile authentication service in Nigeria: An assessment of community pharmacists' acceptance and providers' views of successes and challenges of deployment. Pharmacy practice. 2019;17(2):1449.

226. Pande T, Saravu K, Temesgen Z, Seyoum A, Rai S, Rao R, et al. Evaluating clinicians' user experience and acceptability of LearnTB, a smartphone application for tuberculosis in India. mHealth. 2017;3:30.

227. Peek J, Hay K, Hughes P, Kostellar A, Kumar S, Bhikoo Z, et al. Feasibility and Acceptability of a Smoking Cessation Smartphone App (My QuitBuddy) in Older Persons: Pilot Randomized Controlled Trial. JMIR formative research. 2021;5(4):e24976.

228. Petersen CL, Halter R, Kotz D, Loeb L, Cook S, Pidgeon D, et al. Using Natural Language Processing and Sentiment Analysis to Augment Traditional User-Centered Design: Development and Usability Study. JMIR mHealth and uHealth. 2020;8(8):e16862.

229. Phillips S, Kanter J, Mueller M, Gulledge A, Ruggiero K, Johnson M, et al. Feasibility of an mHealth self-management intervention for children and adolescents with sickle cell disease and their families. Translational behavioral medicine. 2021;11(3):724-32.

230. Portenhauser AA, Terhorst Y, Schultchen D, Sander LB, Denkinger MD, Stach M, et al. Mobile Apps for Older Adults: Systematic Search and Evaluation Within Online Stores. JMIR aging. 2021;4(1):e23313.

231. Pulantara IW, Parmanto B, Germain A. Development of a Just-in-Time Adaptive mHealth Intervention for Insomnia: Usability Study. JMIR human factors. 2018;5(2):e21.

232. Schutte JL, Gales S, Filipponi A, Saptono A, Bambang P, McCue M. Evaluation of a Telerehabilitation System for Community-Based Rehabilitation. Int J Telerehabil. 2012;4(1):25-32.

233. Quan AML, Stiell I, Perry JJ, Paradis M, Brown E, Gignac J, et al. Mobile Clinical Decision Tools Among Emergency Department Clinicians: Web-Based Survey and Analytic Data for Evaluation of The Ottawa Rules App. JMIR mHealth and uHealth. 2020;8(1):e15503.

234. Parasuraman A, Colby CL. An Updated and Streamlined Technology Readiness Index:TRI 2.0. Journal of Service Research. 2015;18(1):59-74.

235. Quinn CC, Staub S, Barr E, Gruber-Baldini A. Mobile Support for Older Adults and Their Caregivers: Dyad Usability Study. JMIR aging. 2019;2(1):e12276.

236. Rajkumar A, Vulpi F, Bethi SR, Raghavan P, Kapila V. Usability study of wearable inertial sensors for exergames (WISE) for movement assessment and exercise. mHealth. 2021;7:4.

237. Ramsey RR, Caromody JK, Voorhees SE, Warning A, Cushing CC, Guilbert TW, et al. A Systematic Evaluation of Asthma Management Apps Examining Behavior Change Techniques. The journal of allergy and clinical immunology In practice. 2019;7(8):2583-91.

238. Regmi K, Kassim N, Ahmad NH, Tuah NA. Assessment of content, quality and compliance of the STaR mobile application for smoking cessation. Tobacco prevention & cessation. 2017;3:120.

239. Reyes A, Qin P, Brown CA. A standardized review of smartphone applications to promote balance for older adults. Disability and rehabilitation. 2018;40(6):690-6.

240. Richardson B, Dol J, Rutledge K, Monaghan J, Orovec A, Howie K, et al. Evaluation of Mobile Apps Targeted to Parents of Infants in the Neonatal Intensive Care Unit: Systematic App Review. JMIR mHealth and uHealth. 2019;7(4):e11620.

241. Roberts AE, Davenport TA, Wong T, Moon HW, Hickie IB, LaMonica HM. Evaluating the quality and safety of health-related apps and e-tools: Adapting the Mobile App Rating Scale and developing a quality assurance protocol. Internet interventions. 2021;24:100379.

242. Rodante DE, Kaplan MI, Olivera Fedi R, Gagliesi P, Pascali A, José Quintero PS, et al. CALMA, a Mobile Health Application, as an Accessory to Therapy for Reduction of Suicidal and Non-Suicidal Self-Injured Behaviors: A Pilot Cluster Randomized Controlled Trial. Archives of suicide research : official journal of the International Academy for Suicide Research. 2020:1-18.

243. Schrepp M, Hinderks A, Thomaschewski J. Design and Evaluation of a Short Version of the User Experience Questionnaire (UEQ-S). International Journal of Interactive Multimedia and Artificial Intelligence. 2017;4:103.

244. Rodrigues AT, Sousa CT, Pereira J, Figueiredo IV, Lima TM. Mobile Applications (Apps) to Support the Hepatitis C Treatment: A Systematic Search in App Stores. Therapeutic innovation & regulatory science. 2021;55(1):152-62.

245. Romero RL, Kates F, Hart M, Ojeda A, Meirom I, Hardy S. Quality of Deaf and Hard-of-Hearing Mobile Apps: Evaluation Using the Mobile App Rating Scale (MARS) With Additional Criteria From a Content Expert. JMIR mHealth and uHealth. 2019;7(10):e14198.

246. Rudolf I, Pieper K, Nolte H, Junge S, Dopfer C, Sauer-Heilborn A, et al. Assessment of a Mobile App by Adolescents and Young Adults With Cystic Fibrosis: Pilot Evaluation. JMIR mHealth and uHealth. 2019;7(11):e12442.

247. AttrakDiff. AttrakDiff Questionnaire [Available from: <http://attrakdiff.de>.

248. Salazar A, de Sola H, Failde I, Moral-Munoz JA. Measuring the Quality of Mobile Apps for the Management of Pain: Systematic Search and Evaluation Using the Mobile App Rating Scale. JMIR mHealth and uHealth. 2018;6(10):e10718.

249. Salehinejad S, Niakan Kalhori SR, Hajesmaeel Gohari S, Bahaadinbeigy K, Fatehi F. A review and content analysis of national apps for COVID-19 management using Mobile Application Rating Scale (MARS). Informatics for health & social care. 2021;46(1):42-55.

250. Sandhu H, Wilson K, Reed N, Mihailidis A. A Mobile Phone App for the Self-Management of Pediatric Concussion: Development and Usability Testing. JMIR human factors. 2019;6(2):e12135.

251. Albert W, Tullis T. Measuring the user experience: collecting, analyzing, and presenting usability metrics: Newnes; 2013.

252. Santo K, Richtering SS, Chalmers J, Thiagalingam A, Chow CK, Redfern J. Mobile Phone Apps to Improve Medication Adherence: A Systematic Stepwise Process to Identify High-Quality Apps. JMIR mHealth and uHealth. 2016;4(4):e132.

253. Satre DD, Ly K, Wamsley M, Curtis A, Satterfield J. A Digital Tool to Promote Alcohol and Drug Use Screening, Brief Intervention, and Referral to Treatment Skill Translation: A Mobile App Development and Randomized Controlled Trial Protocol. JMIR research protocols. 2017;6(4):e55.

254. Scherr TF, DeSousa J, Moore C, Hardcastle A, Wright DW. App Usage and Usability Impressions of a Barcode-Based Digital Contact Tracing Platform for COVID-19: Survey Study. JMIR public health and surveillance. 2021;7(3).

255. Schmidt M, Fisher AP, Sensenbaugh J, Ling B, Rietta C, Babcock L, et al. User experience (re)design and evaluation of a self-guided, mobile health app for adolescents with mild Traumatic Brain Injury. Journal of formative design in learning. 2020;4(2):51-64.

256. Schnall R, Cho H, Liu J. Health Information Technology Usability Evaluation Scale (Health-ITUES) for Usability Assessment of Mobile Health Technology: Validation Study. JMIR mHealth and uHealth. 2018;6(1):e4.

257. Schoeppe S, Alley S, Rebar AL, Hayman M, Bray NA, Van Lippevelde W, et al. Apps to improve diet, physical activity and sedentary behaviour in children and adolescents: a review of quality, features and behaviour change techniques. The international journal of behavioral nutrition and physical activity. 2017;14(1):83.

258. Sedlmayr B, Schöffler J, Prokosch HU, Sedlmayr M. User-centered design of a mobile medication management. Informatics for health & social care. 2019;44(2):152-63.

259. Segura-Sampedro JJ, Rivero-Belenchón I, Pino-Díaz V, Rodríguez Sánchez MC, Pareja-Ciuró F, Padillo-Ruiz J, et al. Feasibility and safety of surgical wound remote follow-up by smart phone in appendectomy: A pilot study. Annals of medicine and surgery (2012). 2017;21:58-62.

260. Yip M, Chang AM, Chan J, Mackenzie AE. Development of the Telemedicine Satisfaction Questionnaire to evaluate patient satisfaction with telemedicine: a preliminary study. Journal of telemedicine and telecare. 2003;9(1):46-50.

261. Sengupta A, Beckie T, Dutta K, Dey A, Chellappan S. A Mobile Health Intervention System for Women With Coronary Heart Disease: Usability Study. JMIR formative research. 2020;4(6):e16420.

262. Sereda M, Smith S, Newton K, Stockdale D. Mobile Apps for Management of Tinnitus: Users' Survey, Quality Assessment, and Content Analysis. JMIR mHealth and uHealth. 2019;7(1):e10353.

263. Setiawan IMA, Zhou L, Alfikri Z, Saptono A, Fairman AD, Dicianno BE, et al. An Adaptive Mobile Health System to Support Self-Management for Persons With Chronic Conditions and Disabilities: Usability and Feasibility Studies. JMIR formative research. 2019;3(2):e12982.

264. Sevilla-Gonzalez MDR, Moreno Loaeza L, Lazaro-Carrera LS, Bourguet Ramirez B, Vázquez Rodríguez A, Peralta-Pedrero ML, et al. Spanish Version of the System Usability Scale for the Assessment of Electronic Tools: Development and Validation. JMIR human factors. 2020;7(4):e21161.

265. Shalan A, Abdulrahman A, Habli I, Tew G, Thompson A. YORwalK: Desiging a Smartphone Exercise Application for People with Intermittent Claudication. Studies in health technology and informatics. 2018;247:311-5.

266. Bangor A, Kortum PT, Miller JT. An empirical evaluation of the system usability scale. Intl Journal of Human–Computer Interaction. 2008;24(6):574-94.

267. Shang J, Wei S, Jin J, Zhang P. Mental Health Apps in China: Analysis and Quality Assessment. JMIR mHealth and uHealth. 2019;7(11):e13236.

268. Sharif MO, Alkadhimi A. Patient focused oral hygiene apps: an assessment of quality (using MARS) and knowledge content. British dental journal. 2019;227(5):383-6.

269. Siddique AB, Krebs M, Alvarez S, Greenspan I, Patel A, Kinsolving J, et al. Mobile Apps for the Care Management of Chronic Kidney and End-Stage Renal Diseases: Systematic Search in App Stores and Evaluation. JMIR mHealth and uHealth. 2019;7(9):e12604.

270. Sood R, Stoehr JR, Janes LE, Ko JH, Dumanian GA, Jordan SW. Cell Phone Application to Monitor Pain and Quality of Life in Neurogenic Pain Patients. Plastic and reconstructive surgery Global open. 2020;8(4):e2732.

271. Zhou L, Bao J, Setiawan IMA, Saptono A, Parmanto B. The mHealth App Usability Questionnaire (MAUQ): Development and Validation Study. JMIR mHealth and uHealth. 2019;7(4):e11500.

272. Soomro N, Chhaya M, Soomro M, Asif N, Saurman E, Lyle D, et al. Design, Development, and Evaluation of an Injury Surveillance App for Cricket: Protocol and Qualitative Study. JMIR mHealth and uHealth. 2019;7(1):e10978.

273. Strandell-Laine C, Leino-Kilpi H, Löyttyniemi E, Salminen L, Stolt M, Suomi R, et al. A process evaluation of a mobile cooperation intervention: A mixed methods study. Nurse education today. 2019;80:1-8.

274. Stütz T, Emsenhuber G, Huber D, Domhardt M, Tiefengrabner M, Oostingh GJ, et al. Mobile Phone-Supported Physiotherapy for Frozen Shoulder: Feasibility Assessment Based on a Usability Study. JMIR rehabilitation and assistive technologies. 2017;4(2):e6.

275. Szajna B. Empirical evaluation of the revised technology acceptance model. Management science. 1996;42(1):85-92.

276. Symsack A, Gaunaurd I, Thaper A, Springer B, Bennett C, Clemens S, et al. Usability Assessment of the Rehabilitation Lower-limb Orthopedic Assistive Device by Service Members and Veterans With Lower Limb Loss. Military medicine. 2021;186(3-4):379-86.

277. Talwar D, Yeh YL, Chen WJ, Chen LS. Characteristics and quality of genetics and genomics mobile apps: a systematic review. European journal of human genetics : EJHG. 2019;27(6):833-40.

278. Tan R, Cvetkovski B, Kritikos V, O'Hehir RE, Lourenço O, Bousquet J, et al. Identifying an effective mobile health application for the self-management of allergic rhinitis and asthma in Australia. The Journal of asthma : official journal of the Association for the Care of Asthma. 2020;57(10):1128-39.

279. Teo CH, Ng CJ, Lo SK, Lim CD, White A. A Mobile Web App to Improve Health Screening Uptake in Men (ScreenMen): Utility and Usability Evaluation Study. JMIR mHealth and uHealth. 2019;7(4):e10216.

280. Terhorst Y, Messner EM, Schultchen D, Paganini S, Portenhauser A, Eder AS, et al. Systematic evaluation of content and quality of English and German pain apps in European app stores. Internet interventions. 2021;24:100376.

281. Tinschert P, Jakob R, Barata F, Kramer JN, Kowatsch T. The Potential of Mobile Apps for Improving Asthma Self-Management: A Review of Publicly Available and Well-Adopted Asthma Apps. JMIR mHealth and uHealth. 2017;5(8):e113.

282. Tonheim AN, Babic A. User Evaluation of a Multiple Sclerosis Self-Management Mobile Application. Studies in health technology and informatics. 2018;251:233-6.

283. Torbjørnsen A, Småstuen MC, Jenum AK, Årsand E, Ribu L. The Service User Technology Acceptability Questionnaire: Psychometric Evaluation of the Norwegian Version. JMIR human factors. 2018;5(4):e10255.

284. Hirani SP, Rixon L, Beynon M, Cartwright M, Cleanthous S, Selva A, et al. Quantifying beliefs regarding telehealth: development of the whole systems demonstrator service user technology acceptability questionnaire. Journal of telemedicine and telecare. 2017;23(4):460-9.

285. Ureña R, Chiclana F, Gonzalez-Alvarez A, Herrera-Viedma E, Moral-Munoz JA. m-SFT: A Novel Mobile Health System to Assess the Elderly Physical Condition. Sensors (Basel, Switzerland). 2020;20(5).

286. Van Cleave JH, Fu MR, Bennett AV, Persky MS, Li Z, Jacobson A, et al. The development, usability, and reliability of the Electronic Patient Visit Assessment (ePVA) for head and neck cancer. mHealth. 2019;5:21.

287. Vélez O, Okyere PB, Kanter AS, Bakken S. A usability study of a mobile health application for rural Ghanaian midwives. Journal of midwifery & women's health. 2014;59(2):184-91.

288. Virani A, Duffett-Leger L, Letourneau N. Parenting apps review: in search of good quality apps. mHealth. 2019;5:44.

289. Wang EH, Zhou L, Chen SK, Hill K, Parmanto B. An mHealth Platform for Supporting Clinical Data Integration into Augmentative and Alternative Communication Service Delivery: User-Centered Design and Usability Evaluation. JMIR rehabilitation and assistive technologies. 2018;5(2):e14.

290. Wang X, Markert C, Sasangohar F. Investigating Popular Mental Health Mobile Application Downloads and Activity During the COVID-19 Pandemic. Human factors. 2021:18720821998110.

291. Wang Y, Wang Y, Greene B, Sun L. An analysis and evaluation of quality and behavioral change techniques among physical activity apps in China. International journal of medical informatics. 2020;133:104029.

292. Ware P, Dorai M, Ross HJ, Cafazzo JA, Laporte A, Boodoo C, et al. Patient Adherence to a Mobile Phone-Based Heart Failure Telemonitoring Program: A Longitudinal Mixed-Methods Study. JMIR mHealth and uHealth. 2019;7(2):e13259.

293. Venkatesh V, Thong JY, Xu X. Consumer acceptance and use of information technology: extending the unified theory of acceptance and use of technology. MIS quarterly. 2012:157-78.

294. Weekly T, Walker N, Beck J, Akers S, Weaver M. A Review of Apps for Calming, Relaxation, and Mindfulness Interventions for Pediatric Palliative Care Patients. Children (Basel, Switzerland). 2018;5(2).

295. White BK, Martin A, White JA, Burns SK, Maycock BR, Giglia RC, et al. Theory-Based Design and Development of a Socially Connected, Gamified Mobile App for Men About Breastfeeding (Milk Man). JMIR mHealth and uHealth. 2016;4(2):e81.

296. Wilson H, Stoyanov SR, Gandabhai S, Baldwin A. The Quality and Accuracy of Mobile Apps to Prevent Driving After Drinking Alcohol. JMIR mHealth and uHealth. 2016;4(3):e98.

297. Winoker JS, Koo K, Huang MM, Bhanji Y, Matlaga BR. Systematic Evaluation of Smartphone Applications for the Medical Management of Nephrolithiasis. Journal of endourology. 2021.

298. Woods LS, Duff J, Roehrer E, Walker K, Cummings E. Patients' Experiences of Using a Consumer mHealth App for Self-Management of Heart Failure: Mixed-Methods Study. JMIR human factors. 2019;6(2):e13009.

299. Woodworth GE, Marty AP, Tanaka PP, Ambardekar AP, Chen F, Duncan MJ, et al. Development and Pilot Testing of Entrustable Professional Activities for US Anesthesiology Residency Training. Anesthesia and analgesia. 2021.

300. Ybarra ML, Prescott T, Mustanski B, Parsons J, Bull SS. Feasibility, Acceptability, and Process Indicators for Guy2Guy, an mHealth HIV Prevention Program for Sexual Minority Adolescent Boys. The Journal of adolescent health : official publication of the Society for Adolescent Medicine. 2019;65(3):417-22.

301. Ybarra ML, Holtrop JS, Prescott TL, Strong D. Process evaluation of a mHealth program: lessons learned from Stop My Smoking USA, a text messaging-based smoking cessation program for young adults. Patient education and counseling. 2014;97(2):239-43.

302. Yoo S, Lim K, Baek H, Jang SK, Hwang GY, Kim H, et al. Developing a mobile epilepsy management application integrated with an electronic health record for effective seizure management. International journal of medical informatics. 2020;134:104051.

303. Zaror C, Espinoza-Espinoza G, Atala-Acevedo C, Muñoz-Millán P, Li Y, Clarke K, et al. Validation and usability of a mobile phone application for epidemiological surveillance of traumatic dental injuries. Dental traumatology : official publication of International Association for Dental Traumatology. 2019;35(1):33-40.

304. Thyvalikakath TP, Schleyer TK, Monaco V. Heuristic evaluation of clinical functions in four practice management systems: a pilot study. The Journal of the American Dental Association. 2007;138(2):209-18.

305. Zeleke AA, Worku AG, Demissie A, Otto-Sobotka F, Wilken M, Lipprandt M, et al. Evaluation of Electronic and Paper-Pen Data Capturing Tools for Data Quality in a Public Health Survey in a Health and Demographic Surveillance Site, Ethiopia: Randomized Controlled Crossover Health Care Information Technology Evaluation. JMIR mHealth and uHealth. 2019;7(2):e10995.

306. Zhong R, Rau PP. A Mobile Phone-Based Gait Assessment App for the Elderly: Development and Evaluation. JMIR mHealth and uHealth. 2020;8(2):e14453.

307. Zhou J, Rau P-LP, Salvendy G. Older Adults’ Text Entry on Smartphones and Tablets: Investigating Effects of Display Size and Input Method on Acceptance and Performance. International Journal of Human–Computer Interaction. 2014;30(9):727-39.

308. Zhou L, DeAlmeida D, Parmanto B. Applying a User-Centered Approach to Building a Mobile Personal Health Record App: Development and Usability Study. JMIR mHealth and uHealth. 2019;7(7):e13194.

309. Zijp TR, Touw DJ, van Boven JFM. User Acceptability and Technical Robustness Evaluation of a Novel Smart Pill Bottle Prototype Designed to Support Medication Adherence. Patient preference and adherence. 2020;14:625-34.
